# Effect of a Low Electrostatic Environment on the Helical Structures of Peptides and Proteins Using Flexible Water Models: An In Silico Study

**DOI:** 10.1021/acsomega.5c06782

**Published:** 2025-11-05

**Authors:** Jorge Alberto Aguilar-Pineda, Jesús Pérez-Aguilar, Minerva González-Melchor

**Affiliations:** Instituto de Física “Luis Rivera Terrazas”, 211699 Benemérita Universidad Autónoma de Puebla, Av San Claudio, Cd Universitaria, Apdo. Postal J-48, Puebla 72570, Mexico

## Abstract

The electrostatic representation of the molecular environment surrounding membrane proteins is a topic that has not been addressed in the field of molecular simulations. The forces produced by such environments play a decisive role in processes such as GPCR activation, molecular recognition between membrane components, and interactions with ligands, directly impacting their dynamics and physiological function. Based on the FBA/ϵ and TIP4P/ϵ_flex_ parameters, we have constructed two new flexible water models to produce low dielectric constants in order to study their effect on the structural properties of protein–membrane complexes. These new low electrostatic water (LEw) models were tested on five helical peptides and two helical-type integral membrane proteins (IMPs) by using molecular dynamics simulations and other *in silico* tools. Our results show that LEw models enhance intramolecular interactions by producing more hydrogen bonds within the protein structures, leading to greater compaction and conservation of their secondary structures. In the case of IMPs, a low electrostatic solvent leads to greater interaction between the transmembrane domains, preventing their opening and structural deformation. Furthermore, although these models increased their interactions with the membrane, an improvement in properties such as thickness, area per lipid, and lateral diffusion was observed. These novel models would enable for a more accurate description and understanding of the various interactions between membrane proteins, potentially leading to the development of more effective drugs targeting these therapeutic targets. Furthermore, this new approach could be applied in the study of more complex membrane models. This work highlights the importance of developing new water models that improve the molecular description of the environment surrounding cell membranes and enable us to generate more reliable computer results.

## Graphical abstract

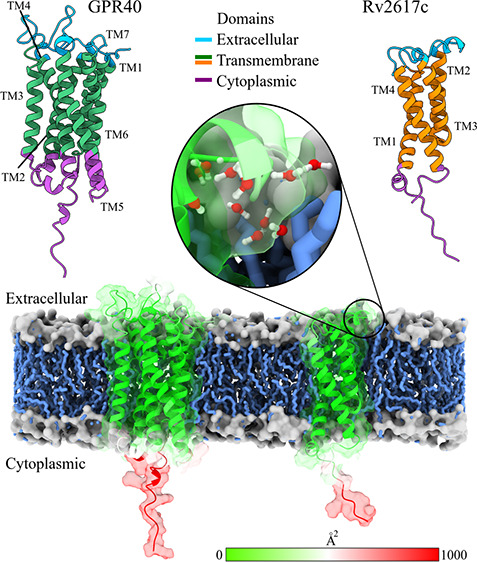

## Introduction

Membrane proteins (MPs) constitute about 30% of the cellular proteome in various eukaryotic organisms, including humans.^1,2^ These proteins fulfill a great diversity of physiological functions ranging from cell recognition, signal transduction, material transport, and enzymatic activity.^3,4^ However, MPs are also associated with the pathology of various diseases (diabetes, cancer, cardiovascular, endocrine, pulmonary, etc.), so 50 to 60% of the drugs currently designed are directed at these proteins considered therapeutic targets.^2^ Despite their importance and the impact in the biomedical area,^5^ the interactions between these membrane proteins and their specific lipid nanoenvironments are not so well characterized. The lack of a molecular picture that accurately represents the environment near the cell membrane could negatively impact the study of biological systems. For example, in drug development, research indicates that the average cost of developing a new drug can reach around one billion dollars, accounting for those that fail in clinical trials.^6^

In this molecular framework, water plays an active role in the different interactions of MPs and all biomolecules, influencing their structure, dynamics, and biological function.^3,7,8^ Various studies indicate that this influence is due to the peculiar properties of a thin layer of water molecules retained by biomolecules, called the hydration layer (HL).^9–12^ Experimental evidence and molecular simulations indicate that the effect of hydration layers can reach up to 2.0 nm in the case of molecular assemblies, such as cell membranes.^9,13,14^ These water layers at the interface of lipid bilayers are so important that they influence the structural stability and fluidity of both lipids and the various components of the lipid matrix (proteins, glycolipids, and cholesterol, among others).^10,15^ Although their influence on the functions and properties of biomolecules has been widely studied, it is little understood why their properties differ from bulk water.^14^

Due to the different interactions established between water and the interface of biomolecules, mainly by hydrogen bonds, the translational and rotational dynamics of water molecules are affected, modifying their degrees of freedom.^9,13,16^ Although it is not entirely clear, this alters several properties that characterize bulk water, among which the dielectric constant stands out.^13,17^ According to Cherepanov et al., at a distance of two nm from the membrane, the value of this constant would be ∼20.^18^ Other properties, such as density and diffusion, are also affected by lipid molecules.^19–21^

Being water, the main component used to represent the molecular environment in molecular simulations, it is common practice to use popular water models that have been parametrized for particular purposes.^22,23^ Nevertheless, the computational efficiency of these models is paramount, and its accurate representation can significantly impact the overall performance and outcomes of the simulations, underscoring the need for models that strike a balance between fidelity and efficiency. Despite their importance, less attention has been paid to the behavior of water models and their effect on the different interactions that take place in the lipid matrix, and only simple models have gained popularity for biomolecular simulation.

As time goes by, new and more sophisticated solvent models can be proposed, which, together with the increase in computational power, allow for a better understanding of interactions at the molecular level and contribute to many areas, such as the development of new drugs, cell recognition, enzyme activation/deactivation, etc.^24^ This is the case with flexible water models, where it has been shown that the inclusion of these degrees of freedom could influence the structural description of biological systems.^23^ These models can influence the study of infrared (IR) and Raman spectra, which aid in the characterization of modified amino acids and proteins that cannot be effectively analyzed with rigid models.^25,26^ Furthermore, they could change the current perspective on protein flexibility, rigidification, and stabilization.^22^

Therefore, the main aim of this work is to analyze the structural and energetic behavior of peptides and proteins in a low electrostatic environment. To achieve this goal, we have developed two flexible water models whose dielectric constant value is close to 20 under physiological conditions (309.65 K and 1 bar of pressure) to simulate the proximity of the lipid membrane. Later, using molecular dynamics (MD) simulations and various in silico tools, we analyzed the effects of these new models on protein structures by calculating the percentage of preservation of secondary structures and intramolecular hydrogen bonds, as well as protein–water interactions. In addition, we analyzed their interactions with lipid molecules and with the protein itself. Our results show that these new models can preserve, to a greater extent, the helical structure in protein configurations, in addition to improving the structural properties of the lipid membrane. These findings raise the need to develop new models that consider the electrostatic effect in these systems to enhance the molecular description of the interactions that occur in cell membrane environments.

## Computational Details

### Reparameterization Procedure of the Water Models

The new interaction and structural parameters of the novel water models were obtained by modifying the H–O–H angle and preserving the original atomic charges from two flexible water models: the FBA/ϵ^27^ and the TIP4P/ϵ_flex_ ^28^ (Table 1). Using MD simulations in the *NPT* ensemble, the target properties were the experimental density of water at ambient pressure (in the 298 to 323 K temperature range at 1 bar of pressure) and the dielectric constant at physiological temperature (309.65 K). The functional form used to calculate the intra- and intermolecular interactions is based on the OPLS/AA force field.^29^ The equilibrium angle HOH was obtained by setting the dielectric constant value at 20 at 309.65 K. Once this property was produced, the Lennard-Jones potential (σ and ϵ) parameters were adjusted to achieve the bulk water density at the same temperature. In this stage, all systems involved 500 water molecules.

**Table 1:** Force Field Parameters of Nonpolarizable Water Models Used as Initial, and Parameters of Those Developed in This Work

model	*r*_OH_, nm	*k*_b_, kJ mol^–1^ nm^–2^	θ, °	*k*_a_, kJ mol^–1^r rad^–2^	ϵ_OO_, kJ mol^–1^	σ_OO_, nm	*q*_H_, *e*
FBA/ϵ	0.10270	300,000	114.70	383.00	0.792324	0.31776	0.4225
FBA_mem_	0.10270	300,000	132.50	383.00	0.792324	0.31616	0.4225
TIP4P/ϵ_flex_	0.09300	157,000	111.50	212.00	0.794032	0.31734	0.5100
TIP4P_mem_	0.09300	157,000	129.0	212.00	0.794032	0.31498	0.5100

### Validation and Assessment of the Original and New Water Models

In order to validate the new interaction parameters, two evaluations were carried out using MD simulations: as a pure component at four different temperatures (277.15, 298.15, 309.65, and 323.15 K) and as a solvating medium for five peptides with a mainly helical structure. Five properties were calculated in the evaluation as a pure component: the dielectric constant, the density, the radial distribution function at 309 K, the self-diffusion coefficient, and the formation of hydrogen bonds. Cubic simulation boxes with 500 water molecules were used for these purposes.

For the second evaluation, the calculation focused on the conservation of helical structures, the number of hydrogen bonds, and the folding or unfolding of the peptide, as measured by its radii of gyration. The structures chosen were amyloid-β (PDB ID: 1IYT^30,31^ ) and four peptides used in treating diabetes mellitus II: exenatide, liraglutide, semaglutide, and tirzepatide (PDB IDs: 7MLL,^32,33^ 4APD,^34^ 7KI0,^35,36^ and 7FIM,^37,38^ respectively). The peptide-water systems were prepared from their experimental structures retrieved from the PBD server. Missing or incomplete residues were built using UCSF Chimera v.1.14 software,^39,40^ and their interaction parameters were obtained through the LigParGen server.^41,42^ Cubic simulation boxes were used to place the peptide at the center of the boxes, ensuring that the minimum distance between the boundaries and the peptide was greater than 2 nm. Then, the systems were solvated with the original and reparametrized water models, and the charge was neutralized by the addition of Cl^–^ and Na^+^ counterions at a concentration of 0.154 M.

### Preparation of Protein–Membrane Complexes

Two membrane proteins, GPR40, and Rv2617, were used to test the performance of the two new water models. The initial three-dimensional structures of the three models were recovered from the AlphaFold database,^43,44^ with the codes AF-O14842-F1 (GPR40),^45^ and AF-I6XER9-F1 (Rv2617c).^46^ A double-layer model was used to simulate the lipid membrane, with 256 dipalmitoylphosphatidylcholine (DPPC) molecules per layer, according to the methodology proposed by Lemkul.^47^ The proteins were embedded in the membrane using the Inflategro methodology.^48^ The transmembrane region of the proteins was determined using the DeepTMHMM server.^49^ The dimensions of the simulation cells along the *x* and *y* axes were set according to the membrane size along those axes. For the *z*-axis, the cell length was set so that the protein surface was no less than 1.5 nm from the cell edges to avoid spurious interactions due to periodic boundary conditions. The systems were solvated, keeping the cell volume constant. Hence, the number of water molecules differs depending on the protein complex and the interaction sites of the water model used. To ensure that no water molecule was within the hydrophobic region of the lipid matrix, defined between the aliphatic chains and the ester group of the lipids, these were eliminated before starting the energy minimization process of the structures.^47^ Finally, Cl^–^ and Na^+^ ions were added to neutralize the electrical charges and mimic the physiological conditions of all systems until reaching an ion concentration of 0.154 M.

### MD Simulations

All molecular dynamics simulations (MDS) were conducted using the GROMACS 2021 package and the OPLS-AA force field.^50–53^ The membrane interaction parameters used in the simulations were obtained from Berger et al.^54^ The first step was to perform an energy minimization using the *steepest descent* algorithm for 50,000 steps, with only the membrane structures and the proteins in vacuum to achieve optimal results. A second energy minimization was performed considering the systems solvated by water and ions. Two equilibrium simulations were then performed in the *NVT* (50 ps) and *NPT* ensembles with position restraints in heavy atoms and a temperature of 323.15 K to ensure stability. The V-rescale thermostat^55^ was used for the *NVT* simulation, while the Nosé–Hoover thermostat^56^ and Parrinello–Rahman barostat^57^ with semi-isotropic pressure coupling were utilized for the *NPT* simulation. The compressibility factor was set at 4.5 × 10^–5^ 1/bar. The *NPT* equilibrium simulation trajectory was 3 ns, saving positions and velocities every nanosecond. These frames then served as initial structures in the production simulations of each replica.

The MD production trajectories were run for 100 (pure component), 200 (peptide-water systems), and 500 ns (protein–membrane systems) in the isobaric–isothermal ensemble at 309.65 K. The trajectories were performed without position restraints, and semi-isotropic pressure coupling was used in the protein–membrane systems. In all simulations, the equations of motion were integrated using a leapfrog integrator with a time step of 1 fs. Periodic boundary conditions (PBCs) were used in the *x*, *y*, and *z* directions. All MD simulations employed a particle mesh Ewald (PME) algorithm^58^ for long-range electrostatics with cubic interpolation and a cutoff of 1.2 nm. Additionally, a linear constraint solver (LINCS)^59^ with all bonds constrained was applied in all MD simulations. The Nosé–Hoover thermostat and the Parrinello–Rahman barostat with a semi-isotropic pressure coupling were used. All production trajectories were saved every ten picoseconds.

### Structure and Data Analysis

Several GROMACS modules were used to obtain statistical results. For water properties as a pure component, *gmx dipoles*, *gmx energy*, *gmx rdf*, and *gmx msd* modules were used to calculate the dielectric constant, density, radial distribution functions, and self-diffusion coefficient values, respectively. In the case of protein complexes, the root-mean-square deviation (RMSD), root-mean-square fluctuation (RMSF), radii of gyration (RG), mean square displacement (MSD), solvent-accessible surface area (SASA) values were computed using *rms*, *rmsf*, *gyrate*, and *sasa* modules. The geometric criteria predetermined by GROMACS were used to calculate hydrogen bonds, i.e., *r* ≤ *r*_HB_ = 0.35 nm and θ < θ_HB_ = 30°.^60^ The VMD software was used to calculate the percentage of HB occupancy along the production trajectories. The structure properties were analyzed using the production MD trajectories of the last 200 ns of each simulation and visualized using the Visual Molecular Dynamics (VMD) and the UCSF Chimera v.1.14 software.^39,40^ The XMGrace software was used to plot graphs.^61^ The energy associated with protein conformation of the different models during MD simulations was visualized using Free energy landscape (FEL) maps. The *gmx sham* module was used to plot these maps, while RMSD and RG were calculated from the atomic position variables concerning their mean structure and the center of mass of the protein, respectively. Finally, figures related to the FEL were built using Wolfram Mathematica 12.1.^62^

## Results and Discussion

### Water as Pure Component

Experimental studies have shown that the electrostatic environment around the membrane is low, influenced mainly by the nonpolar character of lipid molecules and organic cosolvents.^18,63^ In order to evaluate the effect of a low electrostatic environment, we have reparameterized two flexible water models that were developed to reproduce the dielectric constant of water at different temperatures, among other properties: the FBA/ϵ and TIP4P/ϵ_flex_ models.^27,28^ Both models were chosen due to previous work in which we evaluated the ability of these two models to reproduce the structural and energetic properties of the GPR40 receptor.^64^ The results showed that the FBA/ϵ model was the one that best preserved the receptor secondary structures with a distribution of energetic substates close to the minimum energy structure. On the other hand, the TIP4P/ϵ_flex_ model was the worst evaluated in the structural conservation of GPR40, resulting in a greater distribution of energetic substates.

Structure and interaction parameters of the low electrostatic water (LEw) models were achieved using systems of 500 water molecules in MD simulations (Figure 1a). The bending angle was varied to simulate the low electrostatic environment until reaching a dielectric constant value of ϵ = 20. This proposed value agrees with that obtained at a 2 nm distance in the analysis of the dielectric characteristics of the water/membrane interface performed by Cherepanov et al.^18^ Similar values have been reported in other studies.^13,17,63^ σ_LJ_ and ϵ_LJ_ parameters were adjusted to reproduce the bulk water density in the MD simulations. Figure 1b shows the evolution of this property for both the originals and the LEw models through the MD trajectories at physiological temperature (309.65 K). As can be seen, the opening of the bending angle causes the dipole moment of the system to decrease (inset in Figure 1b), leading to systems to an earlier convergence time and reducing the dielectric constant fluctuation. This trend is also observed in the simulations at the different temperatures analyzed, with the TIP4P/ϵ_flex_ model showing the longest convergence time and the most significant fluctuation in this property (Figure S1). Thus, the angle values obtained were 132.5° and 129° for the three and four-site models, respectively (Figure 1c, top left panel).

**Figure 1:**
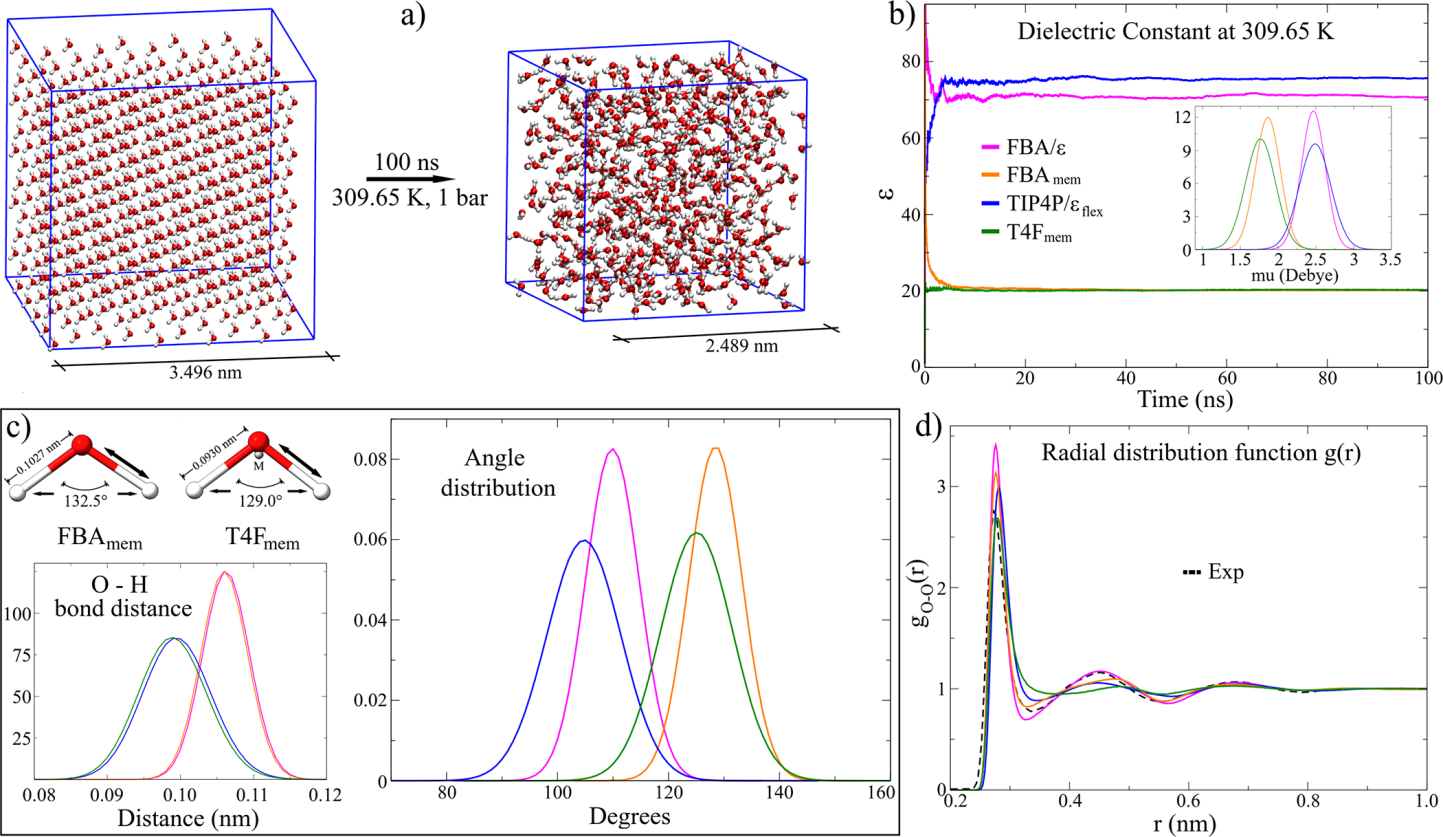
Construction of the low electrostatic models. (a) Simulation conditions. The systems consisted of 500 water molecules, simulated in an *NPT* ensemble at 309.65 K and 1 bar pressure in MD trajectories of 100 ns. (b) Graph of the dielectric constant as a function of simulation time. The convergence of the systems was based on the decrease in fluctuation of this property. The graphs of the dipole moment distribution are shown in the box. (c) Structural parameters obtained in the new proposed models. (d) Oxygen–oxygen pair distribution function for the analyzed models obtained at 298.15 K.

Structurally, the average O–H bond distances of the LEw models are similar to those of the original models (Figure 1c, bond distance distribution plot). However, the three-site model overestimates the experimental value at 300 K by 6.57% (0.1055 vs 0.0990 nm).^65^ In contrast, the four-site model is very close, with a value of 0.0985 nm (0.51% error). Concerning the average bending angle, the obtained values were 128.46° (FBA_mem_) and 125.04° (T4F_mem_), 16.92 and 19.43% more open than the FBA/ϵ and TIP4P/ϵ_flex_ models. Regarding the experimental value for water in the liquid phase (106.56° at 298 K),^66^ the values increased by 22.98 and 19.71%, respectively (Figure 1c). When analyzing the radial distribution function of the oxygen–oxygen pair (*g*_O–O_(*r*)), it is observed that only the T4F_mem_ model underestimates the height of the first experimental peak (2.43%). However, both the probability and its position fit better in the LEw models concerning their original counterparts. In all the analyzed models, the coordination number was close to four. Regarding the second and third peaks, the *g*(*r*) calculated values in both LEw models underestimate the experimental results. Although a shift of ∼0.2 Å is observed in the second peak, the position of the third peak is recovered at 6.89 Å. These results show consistency in the structural properties of the presented models.

In order to obtain a consistent model, three additional properties were evaluated: density, self-diffusion, and hydrogen bonding (HB) capacity. Moreover, these properties were evaluated as a function of four temperatures, namely, 278.15, 298.15, 309.65, and 323.15 K. Figure 2a shows the decrease in the dielectric constant as the temperature increases in the four models evaluated. Parameterized to reproduce this property, the FBA/ϵ and TIP4P/ϵ_flex_ models exhibit maximum errors of 5.17 and 3.91% regarding the experimental values in this temperature range.^27,28^ In contrast, the LEw models show that at these temperatures, the dielectric constant preserves the low polarity environment with very close values, reflected in their slopes: −0.04 for the FBA_mem_ model and −0.08 for the T4F_mem_.

**Figure 2:**
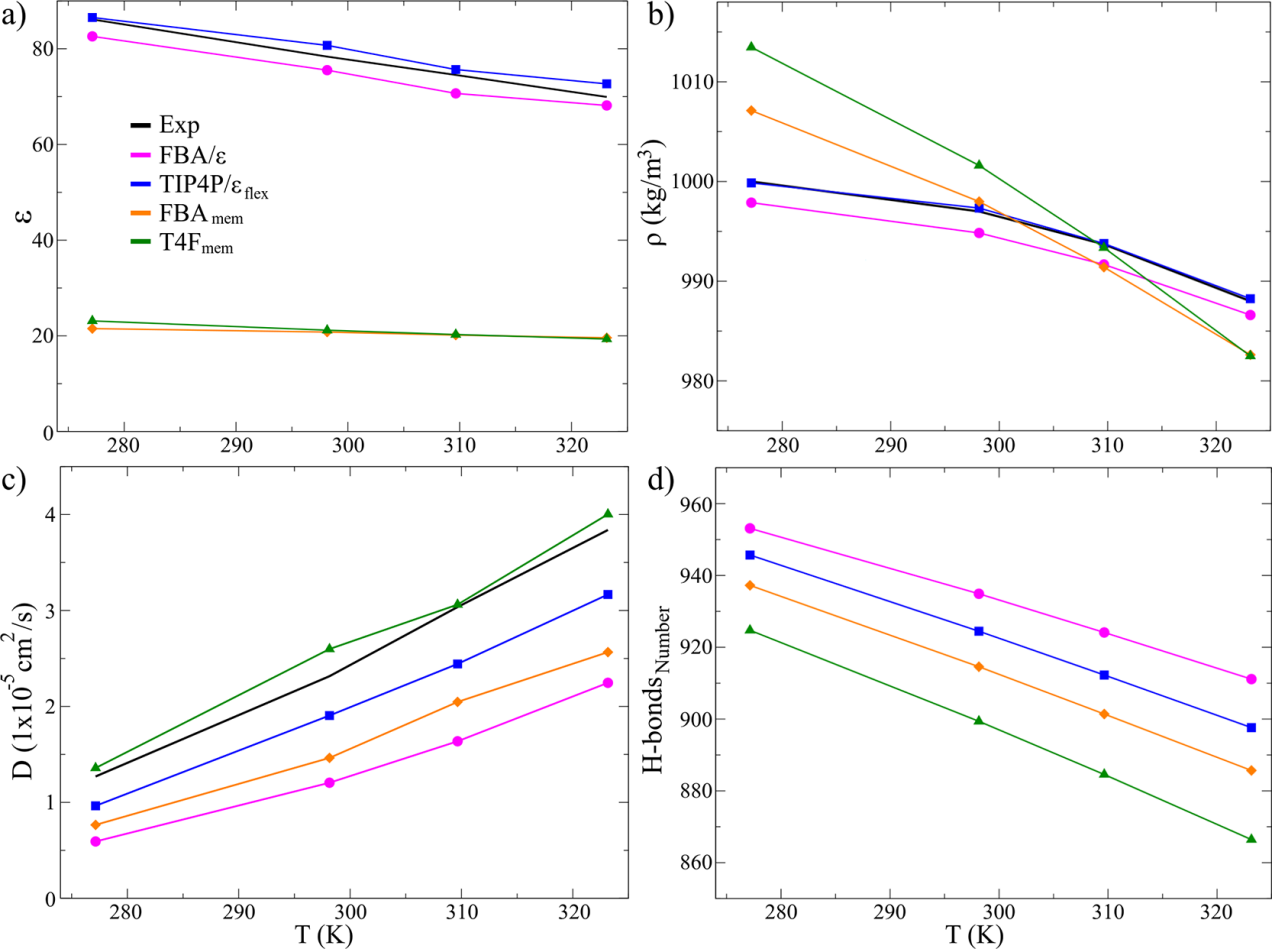
Properties evaluated as a function of temperature: (a) Dielectric constant, (b) density, (c) self-diffusion constant, and (d) hydrogen bond formation.

For the LEw models, the bulk density was the least accurate property to the experimental data­(Figure 2b), fitting only to 309.65 K. At lower temperatures, the values are overestimated (maximum errors of 0.7 and 1.4% for the three- and four-site models at 278.15 K). In comparison, at higher temperatures, the data are underestimated (errors close to 0.5% in both models). Regarding the self-diffusion constants, the LEw models improve this property concerning the original models (Figure 2c). For the FBA_mem_ model, the average error was 35.6%, while for the FBA/ϵ, it was 47.1%. For the four-site models, the T4F_mem_ data was the closest to the experimental values, with an error of 6.1%, being 19.6% the one calculated for the TIP4P/ϵ_flex_.

A crucial property for constructing the LEw models was their ability to form hydrogen bonds. Due to the modification of their dipole moment, an expected effect is the decrease in their intermolecular interactions. As can be seen in Figure 2d, both LEw models tend to form a smaller amount of these bonds at the four temperatures analyzed compared to their original counterparts, with the T4F_mem_ model presenting the lowest HB formation. This decrease is expected to impact the structural properties of the proteins.

### Water Models in Soluble Peptides

A large part of the structural stability of proteins is due to intramolecular hydrogen bonds that preserve their secondary structures and stabilize their tertiary and quaternary structures.^67,68^ One of the main aims of this study is to determine whether the low polarity of the LEw models affects the helical structures in the MPs and, consequently, their three-dimensional configuration. With this in mind, it was decided to conduct an initial assessment of the effect of the low electrostatic environment on five soluble peptides, whose initial secondary structures are mainly composed of α-helices. This step offers a rapid structural characterization of the systems at a low computational cost.^69^ These peptides were amyloid-β (Aβ-42), exenatide, liraglutide, semaglutide, and tirzepatide. In their experimental forms, these peptides exhibit structures with a high percentage of α-helices: 71.4, 55.3, 35.5, 86.7, and 93.1%, respectively (Figure 3a).

**Figure 3:**
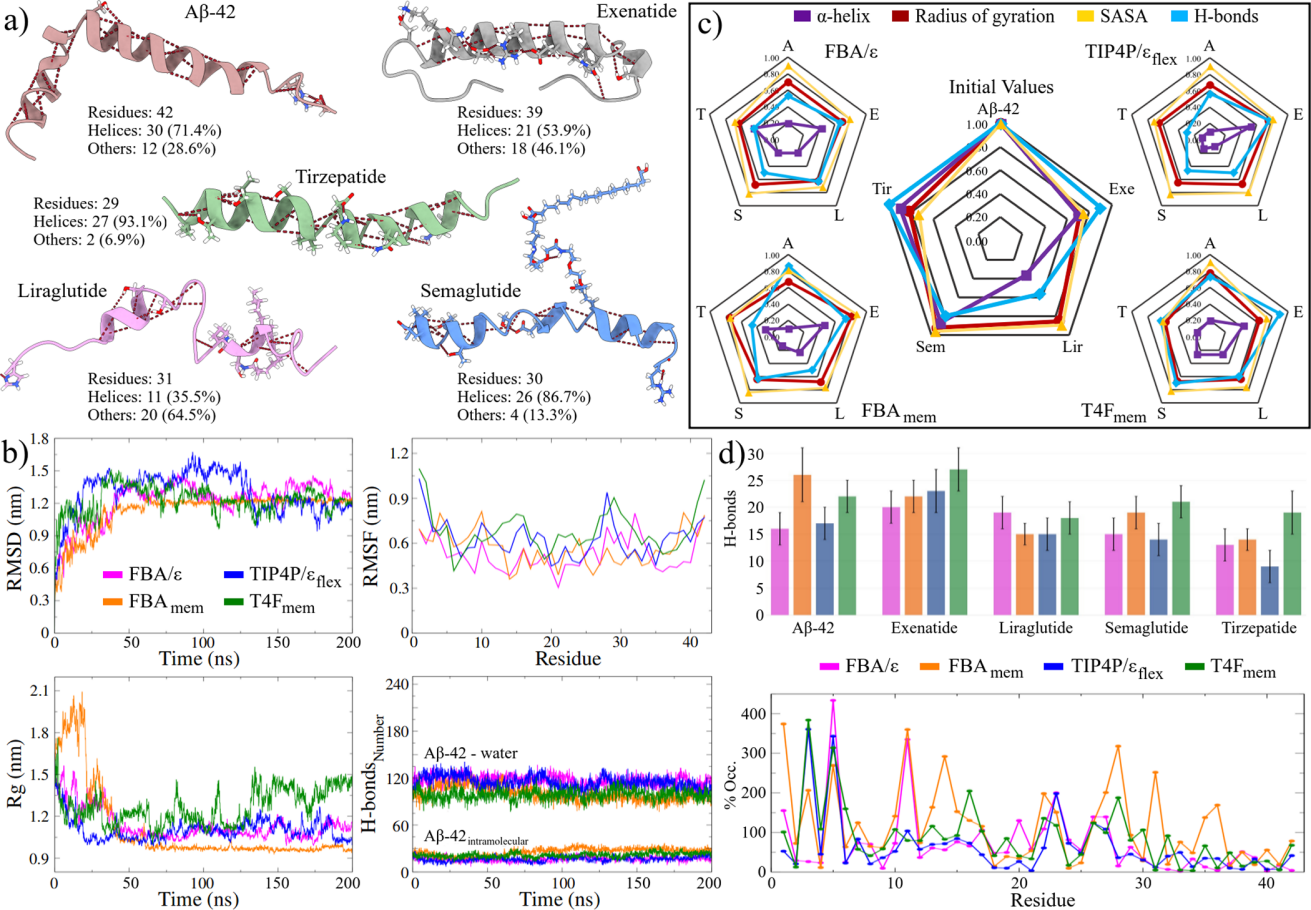
Helical peptides used to assess the LEw models. (a) Experimental structures of the amyloid-β ((PDB ID: 1IYT), exenatide, liraglutide, semaglutide, and tirzepatide peptides retrieved from the Protein Data Bank server (PDB IDs: 7MLL, 4APD, 7KI0,and 7FIM, respectively)). The aliphatic tails of liraglutide and semaglutide were constructed according to the methodology. (b) Stability indicators obtained from the different MD trajectories for the Aβ-42 peptide in the solvated systems. (c) Normalized values of the analyzed structural properties. The MD average values were used to build the radar plots for the solvated systems. (d) Average number of intramolecular H-bonds obtained in the MD simulations (top graph) and % occupancy per residue of the H-bonds obtained for amyloid peptide (bottom graph).

Amyloid-β has been widely studied due to its relationship with the pathophysiology of Alzheimer’s disease, both experimentally and computationally.^70–73^ The other four peptides are analogs of glucagon-like peptide-1 (GLP-1), which are used in treating diabetes, obesity, and other disorders.^74–78^ Plots in Figures 3b and S2 in Supporting Information, illustrate the stability indicators of these peptides calculated in the MD simulations, and their average values of the last 100 ns of the MD trajectories can be consulted in Table S1. Outcomes evidenced that, in general, the convergence of LEw models is comparable to that of their original counterparts. With convergence times around 40 ns and RMSD values close to 0.1 nm, these results are considered characteristic of intrinsically disordered peptides.^79^ In the case of the Aβ-42, similar convergence results have been reported using popular water models (SPCE,^79^ TIP3P,^80–82^ TIP4P^72^) and at different pHs.^73^ Of the five peptides, tirzepatide was the peptide that took the longest to converge (60 ns). Notably, in all models, faster convergence and less fluctuation in the RMSD values were observed for liraglutide and semaglutide, both with lipidated structures. According to experimental studies,^83,84^ lipidation improves structural stability and promotes cellular internalization, half-lives, and pharmacokinetic properties in peptides by acquiring amphiphilic properties.

The effect of lipidation on liraglutide and semaglutide is also observed in the RMSF calculation. The aliphatic chains in these peptides introduce greater fluctuation at residue positions near the lipidated lysine (Lyl26), close to 0.9 nm, with an overall average of 0.55 nm.^72,73^ Despite this, the total RMSF values show the residue fluctuation is reduced in the systems solvated with the LEw models.

Due to decreases in helical structure of the peptides, their preservation was evaluated by four structural properties: percentage conservation of helix structures, intramolecular hydrogen bonding numbers, the total radius of gyration, and the solvent-accessible surface area. Results are summarized in Figure 3c and Table S1. The analyses show exenatide retained the highest percentage of helical structures (0.53% for the TIP4P/ϵ_flex_ model), while the most significant loss was observed in Aβ-42 (up to 90% in the FBA_mem_ and TIP4F/ϵ_flex_ models). This behavior was expected, since it is experimentally known that these peptides tend to form oligomers,^85–89^ and an excess or lack of helices can negatively influence this process.^79^ Similar trends were observed when determining the radii of gyration and SASA in the four water models. Except for exenatide, the other structures tended toward more compact configurations, decreasing their area exposed to the solvent.

Despite the decrease in helical structure, final structures show an average conservation of almost 50% greater for the T4F_mem_ model than the TIP4P/ϵ_flex_ model (Table S1). Furthermore, the FBA/ϵ model had the highest number of residues forming α-helices, with an average of 8.8 residues per peptide. This seems to contrast with experimental studies, which estimate that the helical character decreases with solvent polarity in soluble peptides.^79^

#### Preservation of Secondary Structures and Structure Stability by H-Bonds Interactions

Surprisingly, the property most affected by the interaction with the LEw models was the formation of intramolecular H-bonds (Hb_intra_). As seen in Figure 3c and the top graph of Figure 3d, the average number of Hb_intra_ obtained from the MD trajectories is higher for the LEw models, being more evident in Aβ-42 (FBA_mem_ model) and exenatide (T4F_mem_ model). This increase in intramolecular interaction was mainly due to two factors related to the H-bonding: a decrease in peptide-solvent interactions (Table S1), and an increase in intramolecular H-bond occupancy (Figure 3d, bottom graph, and Figure S3). Furthermore, it is observed that the increase in Hb_intra_ is independent of whether the peptide acquires folded or unfolded conformations (radius of gyration and SASA values). In this context, as in the simulations of pure water, water–water H-bond interactions are lower in the LEw models than in the original models. Interestingly, for the T4F_mem_ model, the H-bond values for both the peptide-water and water–water interactions were the lowest in all systems, which would explain the improvement compared to the TIP4P/ϵ_flex_ model.

On the other hand, H-bond occupancy calculations show that LEw models retain intramolecular interactions longer, especially in the T4F_mem_ model. An important feature is that occupancy values increase in the terminal domains. These regions lose helical structure during simulations, that is, despite the decrease in secondary structure, LEw models promote self-interaction among peptide residues. Moreover, if the average values of this parameter (Table S2) are taken, it is observed that the T4F_mem_ model promoted the greatest intramolecular interaction in the systems, except for the Aβ-42 peptide. Meanwhile, for the FBA_mem_ model, the occupancy was higher than in the FBA/ϵ model in Aβ-42, semaglutide, and tirzepatide peptides. These findings suggest that this parameter could be a target property for new water models focused on studying these systems.

To gain a broader picture of the conformational changes in the peptides, a free energy landscape (FEL) analysis was performed on all MD trajectories (Figures 4a and S4a–d). From this analysis, we extracted the minimum energy structures to verify whether the LEw models followed the tendency to form a greater number of H-bonds than their original counterparts. The results were compared with the final structures (200 ns) obtained for each system (Figures 4b, S4e, and Table S3).

**Figure 4:**
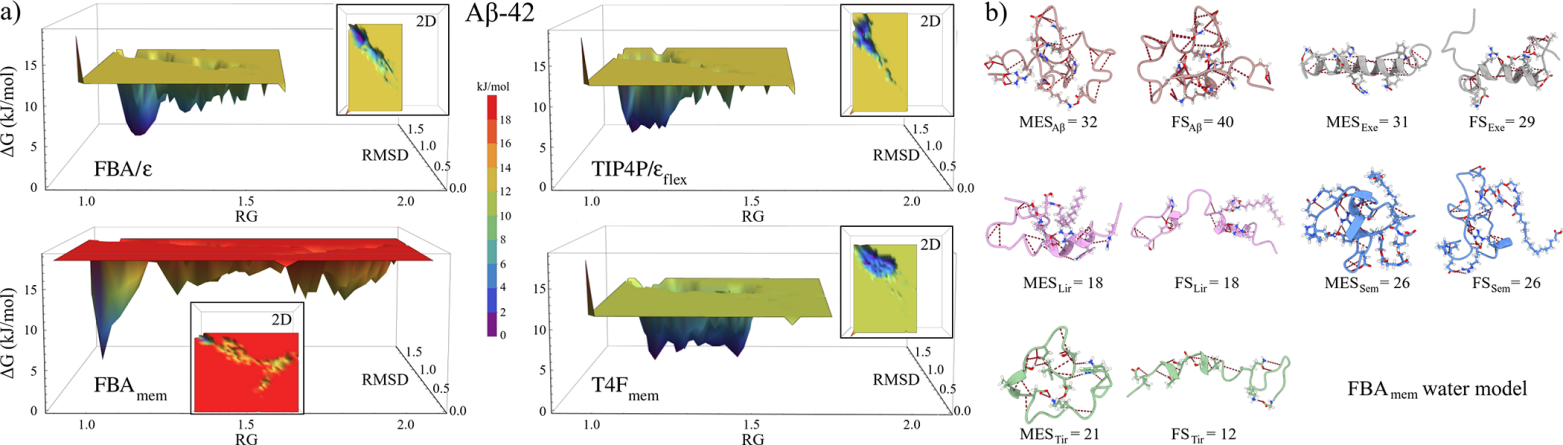
Free energy landscape (FEL) analysis of the MD trajectories of amyloid-β with the four water models studied. (a) 3D plots of the configuration space of each system. The lowest energy configurations are shown in purple and the highest in red. The insets show 2D plots from the top point of view. (b) Minimum energy structures (obtained in FEL analysis), and MD final structures. Both show the number of H-bonds obtained with the ChimeraX v.1.9 ^90^ viewer using the geometric criterion adopted by Gromacs (*r* ≤ 0.35 nm, θ ≤ 35°).

Outcomes show the four water models produce similar sizes in the configurational space (CS), as observed in the FEL area values in Table S3. Except for exenatide, where the CSs in the four-site water models were smaller, the peptides acquire a great diversity of conformations associated with local minimum energy states. This high dispersion of conformations occurs when the helical peptides behave as disordered structures upon losing their helical structure. However, it can be observed that the LEw models present lower energy values in their global minimum energy conformations (”FEL energies” column, Table S3). These minimum energy states suggest the additional stability could be due to the greater H-bond formation in the LEw models.

Analyses of the minimum-energy structures showed a high level of H-bond formation in the structures obtained from the LEw models (Table S3). On average, Hb_intra_ formation for the FBA_mem_ model was 24.4 and 39.8% higher than the FBA/ϵ and TIP4P/ϵ_flex_ models. The most remarkable difference was observed with the T4F_mem_ model, with percentages 50.3 and 68.9% higher than the FBA/ϵ and TIP4P/ϵ_flex_ models.

Despite these results, forming more intramolecular H-bonds did not imply more significant conservation of helical structure in the minimum-energy configurations. Considering an overall average across the five peptides, of the four models, the FBA/ϵ model conserved the highest average percentage of α-helices in the peptides (28.3%). In contrast, the TIP4P/ϵ_flex_ model conserved the least (17.9%). This trend persisted when considering the final structures obtained at 200 ns (Table S1). The only model that increased the α-helix percentage was the T4F_mem_, rising from 20.0 to 23.6% on average.

### Water in Protein–Membrane Systems

Now, how well do LEw models perform as solvents when used in more complex systems involving the presence of a lipid matrix? To explore the electrostatic effect of these models on MPs, we chose two proteins with a predominantly helical structure: the GPR40 receptor (70%) and Rv2617c (71.9%, Figure 5a).^45,46^ The former (300 residues in length) is an MP from the G protein-coupled receptor (GPCR) family, involved in insulin secretion, neurogenesis, and other physiological processes.^64,91,92^ The latter (146 residues) is a probable transmembrane protein related to the bacterium *Mycobacterium tuberculosis* (Mtb) virulence factor, which causes tuberculosis.^73,93–95^

**Figure 5:**
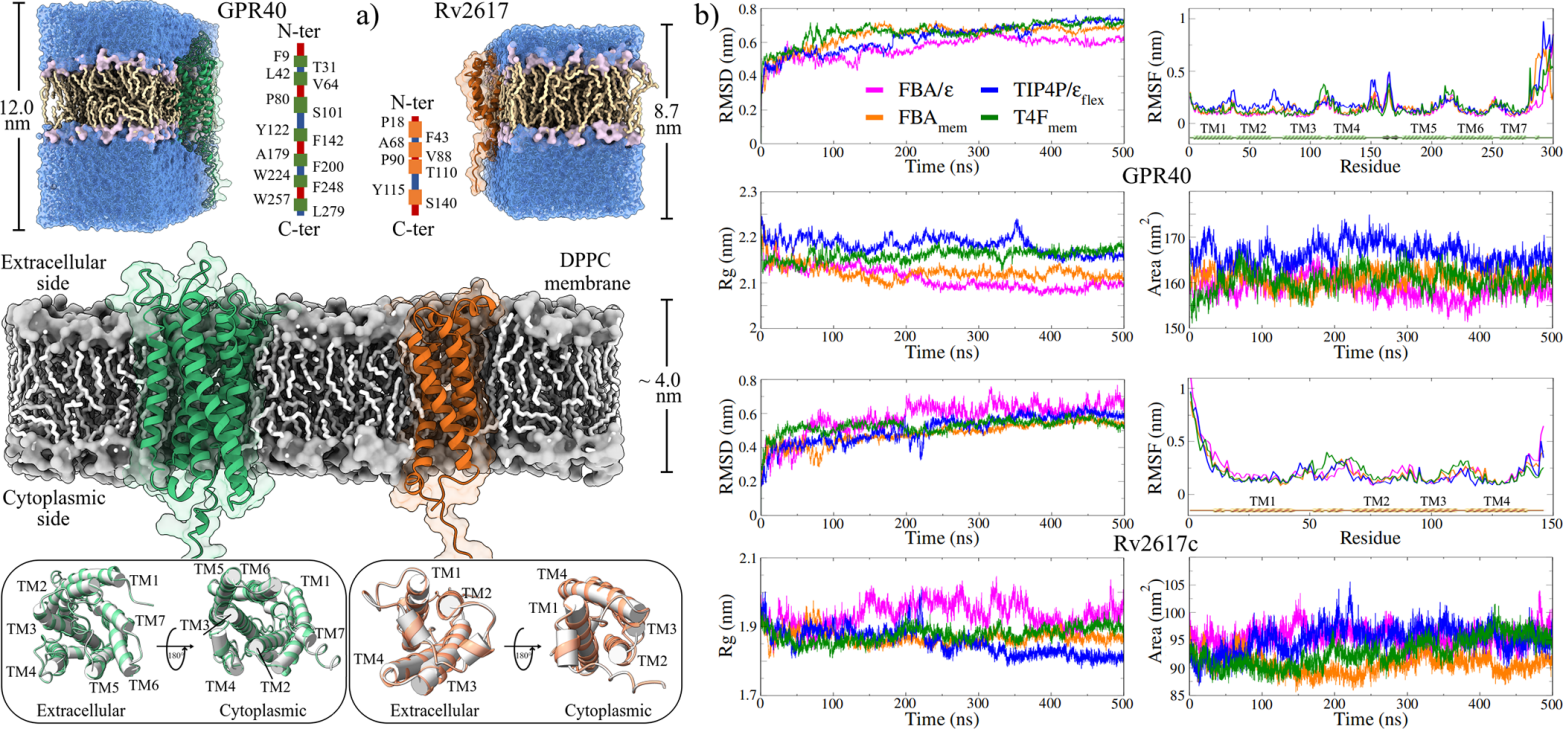
Evaluation of the effect of LEw models on the structural properties of membrane proteins. (a) Construction of the GPR40 and Rv2617c systems embedded in the DPPC lipid bilayer. The *z*-dimension of the simulation cell for the Rv2617c protein is smaller because its extracellular domains are not as exposed. (b) Plots of the stability descriptors used in the MD trajectory analyses. All series correspond to the averaged values of the two replicates performed for each water model.

In order to better understand and read the results, this section will refer to these proteins using the abbreviations G40 and R26. The same color code will be used to represent each water model. Two replicas were built for each system, starting from different positions and velocities to ensure independent results. Each MD trajectory lasted 500 ns at 309.65 K and of 1 bar. The statistical results presented in this section, and subsequent subsections, correspond to the averaged values of both replicas.

#### Structural Stability of the Membrane Proteins in the Water Models

RMSD calculations indicate that the structures of the two MPs attained stable configurations in the MD trajectories. Plots of this descriptor reveal that in the LEw models, the MPs reached an equilibrium plateau before 150 ns. In contrast, in the original models, this equilibrium lasted up to 300 ns (Figure 5b), with slightly larger fluctuations in their values (Table 2), particularly in the systems solvated with the FBA/ϵ model. This stability is reflected in the calculation of other stability indicators, such as the RMSF, the radius of gyration, and the SASA, where the variation of their average values is not significant.

**Table 2:** Average Values of Stability Descriptors of the Studied Systems

			radius of gyration^a^					
water model	RMSD^a^	RMSF^a^	total	*x*_axis_	*y*_axis_	*z*_axis_	SASA^b^	volume/area^c^
GPR40								
FBA/ϵ	0.62 ± 0.04	0.18 ± 0.15	2.09 ± 0.01	1.88 ± 0.01	1.86 ± 0.01	1.32 ± 0.01	158.71 ± 4.03	33.17/17.08
FBA_mem_	0.68 ± 0.01	0.16 ± 0.11	2.12 ± 0.01	1.91 ± 0.01	1.91 ± 0.01	1.30 ± 0.01	161.43 ± 1.84	32.74/17.95
TIP4P/ϵ_flex_	0.70 ± 0.03	0.19 ± 0.13	2.17 ± 0.02	1.95 ± 0.03	1.99 ± 0.02	1.31 ± 0.02	166.39 ± 4.19	33.11/19.02
T4F_mem_	0.70 ± 0.02	0.16 ± 0.10	2.17 ± 0.01	1.96 ± 0.01	1.96 ± 0.01	1.30 ± 0.02	160.43 ± 2.04	32.65/18.07
R2617c								
FBA/ϵ	0.64 ± 0.04	0.17 ± 0.11	1.94 ± 0.03	1.74 ± 0.08	1.73 ± 0.07	1.22 ± 0.05	95.46 ± 1.83	15.95/9.74
FBA_mem_	0.54 ± 0.02	0.16 ± 0.06	1.87 ± 0.01	1.72 ± 0.02	1.73 ± 0.02	1.03 ± 0.03	90.97 ± 1.33	16.17/8.94
TIP4P/ϵ_flex_	0.59 ± 0.03	0.14 ± 0.07	1.83 ± 0.02	1.70 ± 0.03	1.69 ± 0.03	0.99 ± 0.02	94.55 ± 1.77	15.95/9.46
T4F_mem_	0.55 ± 0.02	0.19 ± 0.09	1.89 ± 0.02	1.75 ± 0.02	1.71 ± 0.02	1.08 ± 0.03	95.29 ± 1.78	16.04/9.19

a Values in nanometers.

b Values in square nanometers.

c Values in cubic nanometers/square nanometers. All values were obtained from the last 200 ns of the MD trajectories.

In the G40 systems, RMSF analysis reveals greater fluctuation of the residues outside the lipid matrix, as expected, especially in the terminal region (G280-K300) in all water models. The greatest vibration of the residues was observed in the four-site models, especially with the TIP4P/ϵ_flex_ model, leading to less compact structures of the receptor according to the radius of gyration (*R*_g_) analysis (2.17 compared to 2.09 and 2.11 nm in the three-site models). As shown in Table 2, the values of this descriptor increase along the *x* and *y* axes, which indicates the movement perpendicular to the membrane of the external domains of the receptor. Interestingly, of the two four-site models, the only structure that increased its SASA value compared to its initial value (165.09 nm^2^) was the water-solvated TIP4P/ϵ_flex_ (166.39 nm^2^). These results are consistent with data obtained in a previous study, where this water model exhibited the least compact structures of this receptor.

Similar results are obtained in the R26 systems, where the most significant variation in the values of the stability descriptors is observed in the structures solvated with the FBA/ϵ model. Although the vibrations per residue are close, with the highest RMSF values recorded in the N- and C-terminal domains, the radius of gyration values suggest an opening of their transmembrane domains. In particular, the *R*_g_ values along the *z*-axis with this model show a structure opening of approximately 20% compared to the other systems.

#### The Role of H-Bonds in the Structural Conservation of Helical MPs

Unlike soluble proteins, in membrane environments, the three-dimensional arrangements of proteins do not depend on the hydrophobic effect as the main driving force.^96,97^ For these systems, it has been suggested that the H-bond network and ionic interactions could play a determining role in their conformation and structural stability and, consequently, in their physiological function.^98,99^ In the absence of electrostatic interactions associated with water molecules, intramolecular interactions should be favored, as suggested by the results of the analyzed peptides. To analyze the impact of the LEw modes on the MP-membrane complexes, we used the formation and conservation of Hb_intra_ as well as between TM-TM domains as a stability parameter. Additionally, we measured H-bond formation between protein–solvent (MP-wm), protein–membrane (MP-mb), membrane-solvent (mb-wm), and solvent–solvent (wm-wm) interactions. (Table 3).

**Table 3:** Comparison of Structural Parameters of the Simulated Models^a^

membrane	H-bonds^b^					secondary structure summary^c^ (%)				RMSD^d^
protein	Intra	MP-wm	MP-mb	mb-wm	wm-wm	β-strand	α-helix	3–10 α-helix	other	(Å)
GPR40 (300 residues)										
initial	279					9 (3.0)	205 (68.3)	5 (1.7)	81 (27.0)	-
*TMD*	153					0 (0.0)	140 (88.1)	0 (0.0)	19 (11.9)	
FBA/ϵ	184 ± 5	306 ± 8	34 ± 3	1660 ± 21	70,179 ± 69	2 (0.7)	151 (50.3)	17 (5.7)	130 (43.3)	1.249
*TMD*	110 ± 3	59 ± 3					0 (0.0)	107 (67.3)	7 (4.4)	45 (28.3)
FBA_mem_	187 ± 5	312 ± 8	34 ± 3	1850 ± 21	67,875 ± 74	4 (1.4)	162 (54.0)	13 (4.3)	121 (40.3)	1.024
*TMD*	104 ± 4	74 ± 4					0 (0.0)	107 (67.3)	2 (1.3)	50 (31.4)
TIP4P/ϵ_flex_	162 ± 5	350 ± 9	37 ± 3	1683 ± 20	69,828 ± 78	7 (2.3)	123 (41.0)	15 (5.0)	155 (51.7)	1.208
*TMD*	89 ± 3	92 ± 5				0 (0.0)	91 (57.2)	4 (2.5)	64 (40.3)	
T4F_mem_	183 ± 5	314 ± 9	33 ± 3	1967 ± 23	66,902 ± 91	2 (0.7)	152 (50.7)	9 (3.0)	137 (45.6)	1.096
*TMD*	100 ± 3	79 ± 5				0 (0.0)	109 (68.6)	4 (2.5)	46 (28.9)	
Rv2617c (146 residues)										
initial	133					0 (0.0)	101 (69.2)	4 (2.7)	41 (28.1)	-
*TMD*	89					0 (0.0)	82 (87.2)	0 (0.0)	12 (12.8)	
FBA/ϵ	89 ± 5	160 ± 9	25 ± 3	1683 ± 28	42,217 ± 78	0 (0.0)	72 (49.3)	7 (4.8)	67 (45.9)	1.279
*TMD*	60 ± 3	65 ± 4				0 (0.0)	57 (60.6)	8 (8.5)	29 (30.9)	
FBA_mem_	93 ± 4	156 ± 7	18 ± 2	1877 ± 22	40,767 ± 60	0 (0.0)	81 (55.5)	8 (5.5)	57 (39.0)	0.954
*TMD*	59 ± 3	67 ± 4				0 (0.0)	63 (67.0)	4 (4.3)	27 (28.7)	
TIP4P/ϵ_flex_	83 ± 5	169 ± 9	24 ± 3	1688 ± 29	41,756 ± 81	0 (0.0)	81 (55.5)	3 (2.1)	62 (42.4)	1.286
*TMD*	58 ± 4	73 ± 5				0 (0.0)	59 (62.8)	4 (4.3)	31 (32.9)	
T4F_mem_	92 ± 4	153 ± 6	23 ± 3	2052 ± 23	37,221 ± 69	0 (0.0)	79 (54.1)	12 (8.2)	55 (37.7)	1.190
*TMD*	58 ± 3	68 ± 4				0 (0.0)	65 (69.1)	6 (6.4)	23 (24.5)	

a All values were obtained as averages over two MD simulations, the original and its replica.

b Average number of H-bonds.

c The values indicate the total number of residues that comprise the secondary structure, while the values in parentheses represent the percentages over the total residues of the protein structure.

d Regarding to AlphaFold structure. The RMSD values were calculated considering 272 pairs of residues. TMD: Transmembrane domains. Values obtained by considering only the residues of these domains.

Structurally, the initial configuration of the G40 receptor presents 279 Hb_intra_. Since it is a whole structure without bound ligand, the total number of Hb_intra_ in the AFG40 model is greater than those found in experimental structures, both in the inactive state (254 and 257 for 4phu and 5tzr structures^91,100^ ) and in the active state (225 and 228 for 8eit and 8ejk structures^101^). On the other hand, the AlphaFold configuration of the R26 protein shows 133 Hb_intra_. However, these interactions diminish throughout the simulations, causing the protein to rearrange within the lipid matrix. According to the analysis of the last 200 ns of MD trajectories (Figure 6a), the systems solvated with the TIP4P/ϵ_flex_ model lose the highest percentage of Hb_intra_ interactions, representing 42 and 37% for G40 and R26 MPs, respectively (Table 3, Intra column). For the other models, the loss in these interactions is similar in both MPs, representing a decrease of ∼33%. Nevertheless, the structures of both proteins present greater intramolecular interactions when the LEw models are used as solvents. In the case of the three-site models, the improvement is slight and falls within the statistical error (±5). In contrast, the difference is notable in the four-site models, as the T4F_mem_ model exhibits interactions greater than 7% compared to the original model.

**Figure 6:**
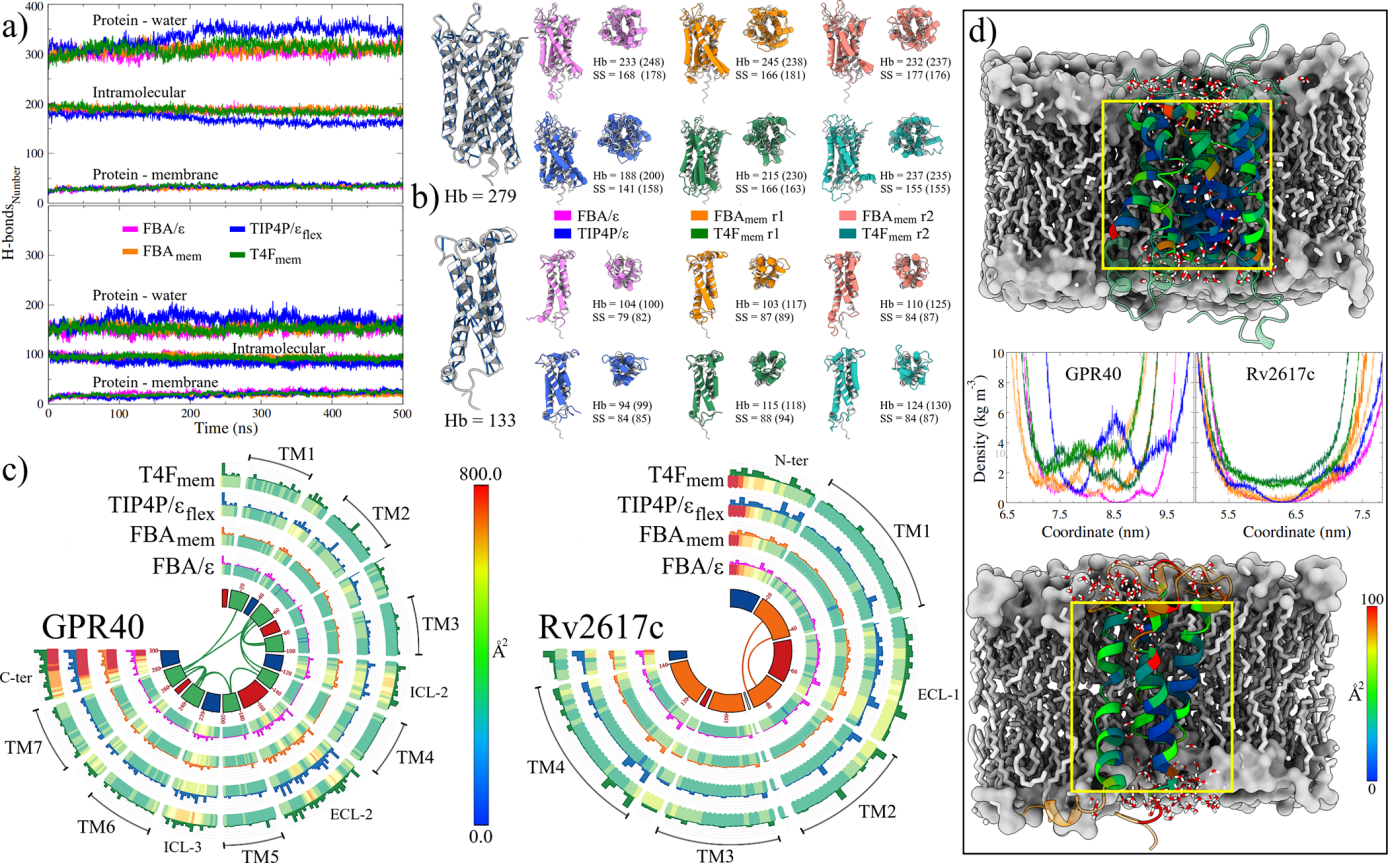
Effect of the water model on the formation, conservation, and occupancy of hydrogen bonds in the MP structures. (a) Average H-bond graphs obtained during MD simulations of the different MP interactions. (b) Intramolecular H-bonds of the initial, lowest-energy, and final conformations of the MPs. The final structures were aligned with those retrieved from the AlphaFold server. Numerical data correspond to the H-bonds and the conserved secondary structure (SS) residues number in the final and lowest-energy conformations (values in parentheses). (c) Average values of the *b*-factor (heat maps) and H-bond occupancy percentages (histograms) of the MP-water interactions, mapped on Circos plots. The color coding of the heat maps is shown in the bar. In the histograms, the colors correspond to the water model used in the analyses, ranging from 0 to 1000%. The lines in the center of the plots depict the intramolecular interactions of the TM domains of each MP in the initial structures. The central circular annulus represents the complete structure of the MPs. The extracellular domains are colored red, the intracellular domains are colored blue, and the TM domains are colored green (G40) and orange (R26). (d) Penetration of water molecules into the core of the MPs. The TM domains of the MPs are colored according to their *B*-factor value. Central graphs show the partial density of the water models analyzed. For the LEw models, results of each simulation are depicted with its replica. Different shades of green were used for the G40 systems, and orange for the R26 systems.

This change in the pattern of intramolecular interactions in the LEw systems impacts the structural properties of the MPs. Using the PDBsum Generate server,^102^ an analysis of the conservation of secondary structures (SS) in the minimum-energy and final conformations was performed (Table 3). Outcomes show that the most significant conservation of helical structures (α-helix + 3–10 α-helix) was obtained using LEw models, which would allow for better structural conservation of the MPs. To corroborate this point, an alignment was performed between the final structures and the AlphaFold models (Figure 6b). RMSD values show that the LEw configurations are closer to the initial structure than those obtained with the original models (Table 3).

These decreases in electrostatic interactions would explain the performance observed with the LEw models and the previously analyzed three-site models.^64^ It is essential to mention that, although the FBA/ϵ model had the lowest interaction with the G40, the FBA_mem_ model exhibited better preservation of the helical structures. On the other hand, the TIP4P/ϵ_flex_ model overestimates this interaction in both MPs compared to the average of the other three models (12.5% in the G40 systems and 9.7% for R26). The decrease in the electrostatic interaction is also observed in their mw-mw interactions (Table 3). As in the pure component simulations, where the decrease was approximately 3% (924 to 901 for the three-site models and 912 to 885 for the four-site models at 309.65 K), H-bond formation is lower in the protein systems. The average reduction in the soluble peptide systems was 3 and 13%, while in the membrane-MP systems it was 3.5 and 12.5%, the former being the value for the FBA models and the latter for the TIP4P models. However, the residues located in the outside regions of the membrane interact most with water molecules, which could influence the findings described above. To address this point, H-bonds and their occupancy in each of the different domains comprising both MPs were analyzed, and results were compared with the b-factors obtained in RMSF calculations (Figure 6c).

As expected, the most significant fluctuations correspond to the extramembrane regions (Figure 6c). Similar fluctuations have been reported in various in silico studies.^64,73,103–107^ In these domains, the most representative occupancy values in protein–water interactions occur. Notably, occupancy is higher in the original models than in the LEw models, and higher in the four-site models than in the three-site models (histograms in Figure 6c). Furthermore, this trend is consistent for both MPs when comparing the values for the ten residues with the highest occupancy (Table S5). For example, at residue E145, located in the ECL-2 domain of G40, the occupancy percentages were 750.2 and 1445.9 for the FBA/ϵ and TIP4P/ϵ_flex_ models, while in the LEw models they were 599.5 (FBA_mem_) and 1146.2 (T4F_mem_). In R26, the residue with the highest occupancy in all four models was D6, located in the N-terminal domain. The values obtained were 702.8 (FBA/ϵ), 573.8 (FBA_mem_), 1430.8 (TIP4P/ϵ_flex_), and 1003.8 (T4F_mem_). Remarkably, histograms show that the residue interactions do not depend on the water model. Those residues with high occupancies were conserved in all the systems analyzed. These findings are relevant because they suggest that the effect of the LEw models is due to the MP-wm interactions being energetically weaker than in the original models, something expected in nonpolar environments.^99^

The regions with the lowest occupancy were the TM domains, which, immersed in the lipid matrix, generate less interaction with water molecules. However, analysis of the density profiles showed penetration of several molecules into the core of the MPs, primarily with the LEw models (Figure 6d). Figure 6c,d show an increase in the *b*-factor values due, to a certain extent, to the presence of water molecules. In contrast, the highest fluctuations in these domains are observed in residues close to the outside of the membrane, where most of the water molecules are concentrated (Figure 6d). Some internal residues showed high values in the occupancy percentages (Table S5).

In this regard, the final configurations of the MPs show that water molecules permeate both protein structures in all models analyzed (Figure S5). In the G40 receptor, water penetration reaches the TM core, especially with LEw models, and increases if the model consists of four sites. Helices TM2, TM3, TM6, and TM7 are primarily involved in this water network extending from the extracellular region to the cytoplasm. Conversely, the R26 protein exhibits lower penetration, likely due to fewer TM domains and a reentrant loop (V88–P90), limiting water interaction. Density profiles (Figure 6d) show that the four-site models permeate with more water molecules, with the LEw models reaching the TM core. This behavior in LEw models could increase TM-water interactions, potentially affecting MP stability by disrupting intramolecular H-bonds, as seen in other studies of GPCRs.^108,109^ Bertalan et al., using the TIP3P water model^110^ and two opioid receptors, noted that dynamic networks of H-bonds within GPCRs are crucial for their activation. However, it could also be an indicator that GPCR-water interactions with this model are energetically favorable due to the high electrostatic effect observed in the calculation of its dielectric constant (94 at 298 K and 1 bar of pressure^111^), causing the breaking of intramolecular H-bonds and resulting in the opening of the structures.

To address this issue, TM-water and TM-TM electrostatic interactions of both MPs, and their occupancy percentages, were calculated. Initial structure of the G40 exhibits 13 H-bonds between its TM domains (Hb_TM_), with TM2 and TM7 having the highest number of H-bonds (six) with three different TM domains. TM2 interacts with TM3 (S51-P89, S58-H86), TM4 (Y44-S123, N47-W131) and TM7 (double interaction D52-N272). Meanwhile, the TM7 domain binds to the TM1 (V269-N23), TM2, and TM6 (R258-Y240, R258-N244, and T264-C236) domains as seen in top panel in Figure 7a. On the other hand, R26 only presents two Hb_TM_, R27-A87 and K41-E79, belonging to the TM1 and TM2 domains (Figures 6c and 7a). Occupancy analyses show that in G40 systems, the T4F_mem_ model performs best, preserving a large portion of the initial Hb_TM_. In the three-site models, both exhibit similar behaviors, with greater conservation in the FBA/ϵ model. Five initial Hb_TM_ obtained values less than 20% in all four models, indicating that these interactions are lost during MD trajectories (center panel in Figure 7a). These interactions were N23–V269, S51–P89, and C236-T264 located near the TM core, and Y240-R258 and N244-R258 in the extracellular region. In the case of R26, both Hb_TM_ obtained values higher than 50%, with values higher than 100% found with the LEw models for the K41-E79 interaction.

**Figure 7:**
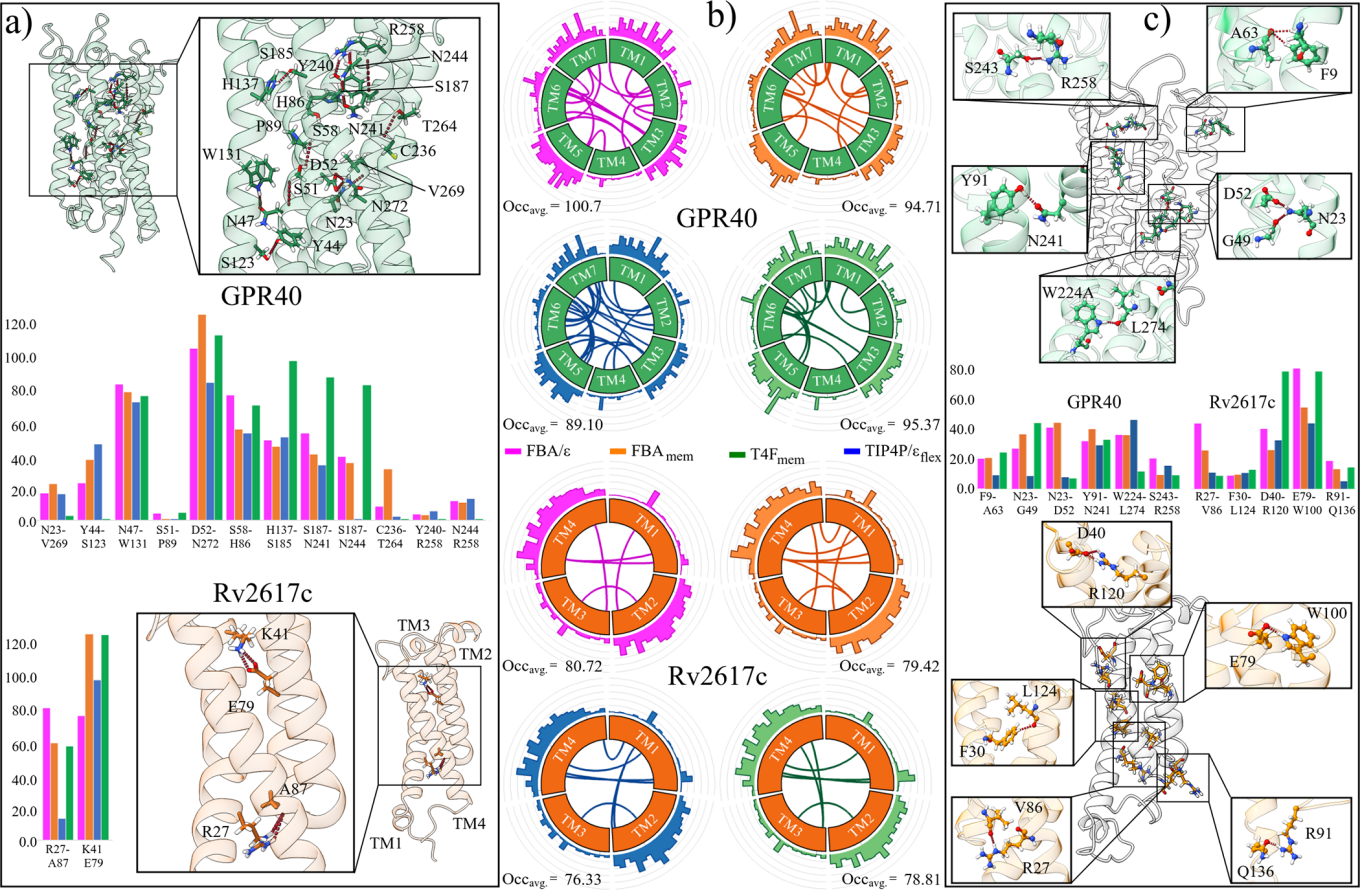
Occupancy and conservation of H-bonds between TM domains. (a) H-bond interactions found in the initial MP configurations (AlphaFold) and their conservation throughout MD simulations. (b) Histograms of the average TM-TM interactions for the analyzed models. Only residues comprising the TM domains are shown. The most significant interactions (>50% occupancy) are shown in the center of each plot. (c) Most significant H-bonds formed during the simulations. The color code for each water model is the same as in previous figures.

As the systems reached structural stability, new interactions between TM domains formed (center lines in the Circos diagrams, Figure 7b and Table S6). The occupancy histograms show higher values in domains TM1, TM3, TM5, and TM7 for G40, and TM2 and TM4 for R26 in the four water models analyzed, being, on average, higher in the FBA/ϵ and T4F_mem_ models (Figure 7b). Although the TIP4P/ϵ_flex_ model presented the greatest TM–TM interaction, its average occupancy values were the lowest in both MPs, being of a more temporal nature. This is demonstrated by the poor conservation of the helical structure of its TM domains (Table 3). Among the most significant interactions are those conserved in all four models and whose occupancy values were greater than 20% (Figure 7c). For G40, six interactions were detected, four of which are located close to the TM core, where the LEw models present higher occupancy values (Figure 7c, top panel). On the other hand, for R26, five interactions were found, which were located along the TM domains. In these systems, the structures solvated with the FBA/ϵ and T4F_mem_ models were the ones that best conserved these interactions (Figure 7c, bottom panel).

#### Effect of Water Models on Lipid Structures

One of the most notable changes in the LEW models was their interaction with lipid molecules, which increased by 11.5% (FBA_mem_) and 19.2% (T4F_mem_, Table 3). This change was reflected in the structural properties of the membrane. For example, lateral lipid diffusion was overestimated by all four models by an order of magnitude, relative to its experimental value at 310 K (0.38 × 10^–7^ cm^2^/s, Table 4 ^112^). This overestimation could be justified because, at 309.65 K, the temperature used in our simulations, DPPC lipids are at phase transition temperatures (310–320 K), where lateral diffusion does not follow a monotonic behavior.^113^ Similar lateral diffusion values have been reported for lipids with other water models measured at 309.65 K.^64,73,103^ Regarding the lateral diffusion of MPs, while the protein–membrane H-bond analysis shows that the number of interactions is similar in the analyzed models (Table 3), the MSD plots exhibit more complex and variable lateral diffusions, with negative slopes at different times along the trajectories. However, in both MPs solvated with the LEw models, the proteins show a slight increase in diffusion, with the R26-T4F_mem_ system being the only one that maintained a monotonically increasing behavior.

**Table 4:** Physical Properties of DPPC Lipid Bilayer at 309.65 K^a^

water	diffusion (× 10^–07^ cm^2^/s)		area per lipid (Å^2^)		bilayer
model	DPPC	protein	up	down	thickness *D*_HH_ (nm)
GPR40					
**Exp.**	0.38 ^112^	0.50 ^114^	51.68 ^115^		3.6–4.4 ^116^
FBA/ϵ	2.73 ± 0.41	0.10 ± 0.15	47.68 ± 0.47	47.42 ± 0.76	4.30 ± 0.02
	2.82 ± 0.36	0.19 ± 0.48	47.99 ± 0.33	47.20 ± 0.36	4.25 ± 0.07
FBA_mem_	3.19 ± 0.12	0.31 ± 0.21	49.48 ± 0.50	48.89 ± 0.61	4.22 ± 0.01
TIP4P/ϵ_flex_	4.17 ± 0.11	0.33 ± 0.10	49.06 ± 0.72	47.43 ± 0.68	4.33 ± 0.01
	3.43 ± 0.09	0.46 ± 0.22	48.81 ± 0.66	47.33 ± 0.71	4.38 ± 0.02
T4F/_mem_	6.56 ± 0.72	0.34 ± 0.01	51.11 ± 0.51	50.12 ± 0.53	4.17 ± 0.01
Rv2617c					
FBA/ϵ	3.02 ± 0.37	0.13 ± 0.06	47.83 ± 0.51	47.72 ± 0.55	4.27 ± 0.31
FBA/_mem_	3.46 ± 0.36	0.14 ± 0.21	49.66 ± 0.48	50.07 ± 0.46	4.19 ± 0.33
TIP4P/ϵ_flex_	5.23 ± 1.10	0.10 ± 0.10	48.07 ± 0.57	48.48 ± 0.55	4.27 ± 0.31
T4F/_mem_	6.84 ± 1.01	2.34 ± 0.40	51.88 ± 0.56	52.35 ± 0.56	4.13 ± 0.35

a The experimental values reported are at 309 K. For the area per lipid, Up is the top layer, and Down is the bottom layer. Values in italics refer to the structural properties of the DPPC membrane reported in our previous work.^64^

Experimental and in silico studies indicate that, without cholesterol, the area per lipid in DPPC models rises to around 64 Å^2^ at 323 K.^113,117,118^ In contrast, at lower temperatures (∼290 K), it decreases to 46 Å^2^, similar to mammalian membranes (42.1 Å^2^) at 303 K.^119^ Walter et al. employed Machine Learning and the TIP3P water model to measure this property across temperatures, finding it to be 51.68 Å^2^ at physiological temperature.^115^ Based on these results, LEw models improved accuracy, with both MPs showing an average percentage error lower than that of the original models (4.17 and 7.77% in the three-site models, and 0.61 and 6.62% in the four-site models).

The last structural parameter analyzed was the thickness of the lipid bilayer. Essential for various functions of MPs and many cellular functions, thickness is a descriptor in the characterization of any membrane model.^120,121^ Experimentally, the thickness of the DPPC membrane at phase transition was determined using temperature-controlled scanning force microscopy.^116^ Measured as the distance between the lipid headgroup phosphates (*D*_H–H_) at a temperature of 309 K, the thickness value was 4.4 nm. Using this value as a reference, it is observed that the LEw models underestimate the thickness, with errors of 4.43% in the FBA_mem_ model and 5.68% in the T4F_mem_.

## Conclusions

One of the main objectives of computational simulations is to essentially reproduce the environmental conditions under which biomolecules perform their physiological functions. However, the choice of water model is often based more on computational cost than on its effect on the molecular environment, as is the case with membrane proteins. These systems experience low-polarity molecular environments, which popular or currently available water models do not adequately reproduce.

In this context, we present the development of two flexible water models with three and four interaction sites, whose target property was to reproduce low-electrostatic environments. The purpose was to explore their potential effects on the helical structures that characterize some membrane proteins, such as GPCRs, where their conservation plays a key role in their biological function. Using five soluble peptides and two MPs with a predominantly helical structure, we have shown that our models improve their structural properties by promoting the formation of intramolecular H-bonds, limiting protein–water interactions. Furthermore, these models showed high interaction with lipid molecules, improving properties such as area per lipid and thickness in a DPPC membrane model.

While the structural properties of proteins and membranes are also influenced by the parameters defined by their force fields, these new water models aim to improve the description of electrostatic interactions produced by the solvent. So far, we have only tested these models using the OPLS-AA force field and the DPPC membrane model developed by Berger et al., with satisfactory results. It would be interesting to apply them to other force fields for proteins and to new membrane models, and to verify their general applicability in these systems.

Despite the limitations of this work, the results open up the possibility that these models can be used to study protein–membrane systems. Their use could improve the structural description and interactions of MPs with the different components of the lipid matrix, leading to the development of more effective drugs. Undoubtedly, there are many questions to be addressed, further explored, and studied at different levels and approaches on this topic. However, we hope that this study will serve as motivation for future work in the development of new water models that seek to reproduce the biological environment in these systems (Table 5).

**Table 5:** Key Resources Table

software or server	source	identifier
Gromacs v.2021	^50^	https://manual.gromacs.org/2021/download.html
VMD	^39^	https://www.ks.uiuc.edu/Research/vmd/
UCSF ChimeraX	^90^	https://www.cgl.ucsf.edu/chimerax/download.html
UCSF Chimera	^40^	https://www.cgl.ucsf.edu/chimera/download.html
RCSB PDB	^122^	https://www.rcsb.org
UniProt	^123^	https://www.uniprot.org
AlphaFold DB	^43,44^	https://alphafold.ebi.ac.uk
DeppTMHMM	^49^	https://dtu.biolib.com/DeepTMHMM

## Supplementary Material



## Data Availability

Structures and files used in molecular dynamics simulations have been deposited at the following electronic addresses:10.17632/bc378f642k.1 A list of software used in this study can be found in the Key Resources Table (Table 5).

## Notes

Evaluation of the dielectric constant of the low-electrostatic water models (LEw models) and the FBA/ϵ and TIP4P/ϵ_*flex*_ models at different temperatures (Figure S1); helical peptides used to assess the LEw models and their stability indicators obtained from the different MD trajectories (Figure S2); occupancy percentages obtained along the MD trajectories (Figure S3); analysis of the free energy landscape (FEL) and intramolecular hydrogen bonds of the helical peptides used in this work (Figure S4); penetration of water molecules into the core of membrane proteins (Figure S5); stability descriptors of solvated peptides (Table S1); average H-bond occupancy of the intramolecular interactions in the analyzed peptides (Table S2); results of the Free Energy Landscape (FEL) analysis (Table S3); average number of H-bonds formed among the TM domains of GPR40 and Rv2617c proteins during MD trajectories (Table S4); top ten residues with the highest occupancy in the MP-water interactions (Table S5); and electrostatic interactions with significant occupancy values (Table S6) (PDF)
