# Supplementary material for: Effect of a Low Electrostatic Environment on the Helical Structures of Peptides and Proteins Using Flexible Water Models: An In Silico Study
Source: ACS Omega. 2025 Nov 5;10(45):54286–301. doi: 10.1021/acsomega.5c06782 (PMC12631355; doi:10.1021/acsomega.5c06782)
Supplement: Supplementary file 1 [file ao5c06782_si_001.pdf]

Effect of a low electrostatic environment on the helical structures of peptides and proteins using flexible water models. An *in silico* study

Jorge Alberto Aguilar-Pineda[a], Jesús Pérez-Aguilar[a], and Minerva González-Melchor[a]*.

[a] Instituto de Física "Luis Rivera Terrazas", Benemérita Universidad Autónoma de Puebla, Av San Claudio, Cd Universitaria, Apdo. Postal J-48, Puebla 72570, México

Keywords: Water model; Integral membrane proteins; Helical peptides; Electrostatic interactions; Lipid membrane.

*Author to whom correspondence should be addressed.
e-mail: minerva@ifuap.buap.mx (M. González-Melchor)

Supplementary Figures

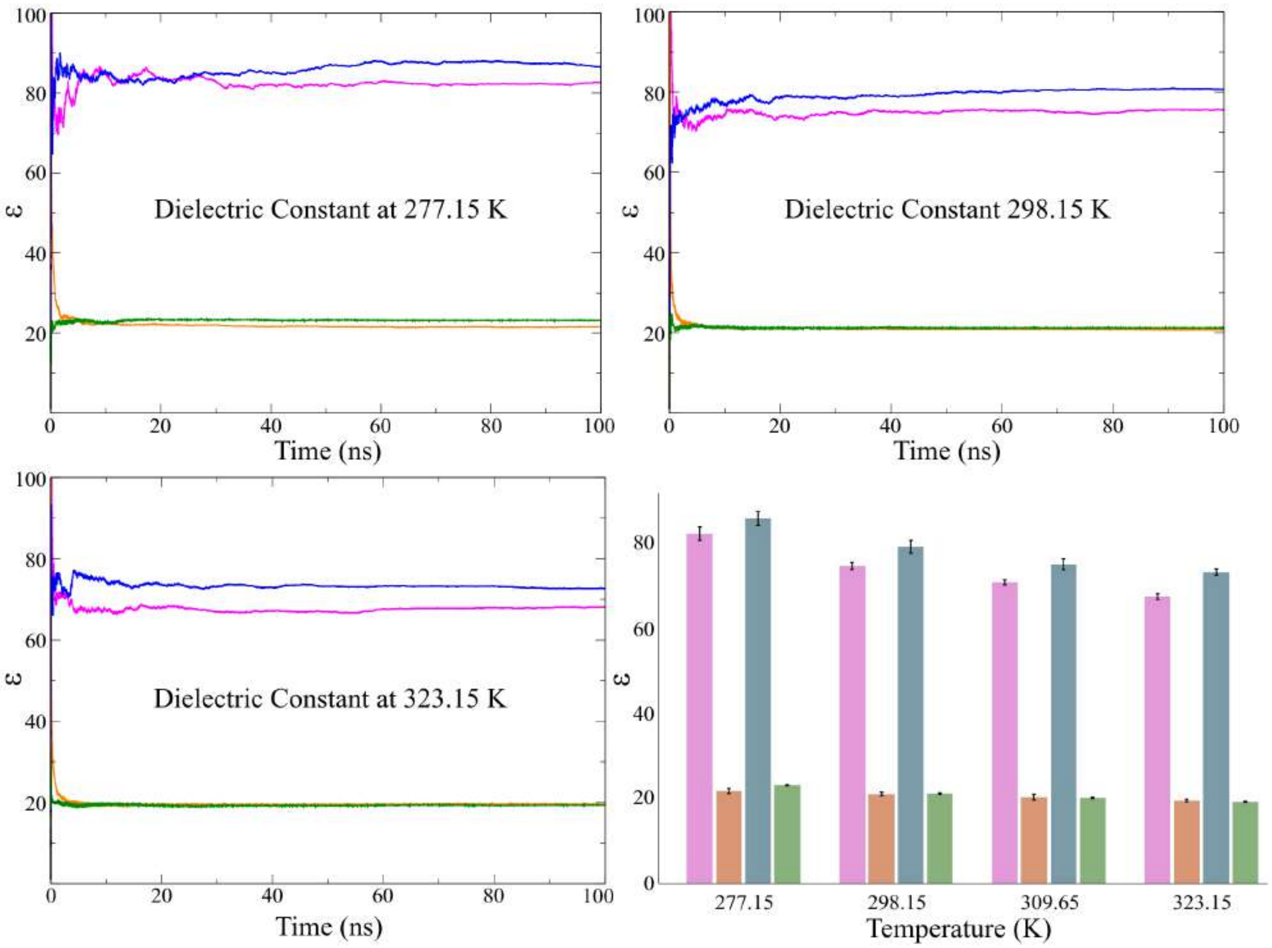

Figure S1. Evaluation of the dielectric constant of the low-electrostatic water models (LEw models) and the FBA/ϵ and TIP4P/ϵ_{flex} models at different temperatures. The systems consisted of 500 water molecules, simulated in an NPT ensemble at 1 bar pressure, with MD trajectories of 100 ns. The LEW models, FBA_{mem} and $T4F_{mem}$, are shown in orange and green, while the original models are shown in magenta and blue.

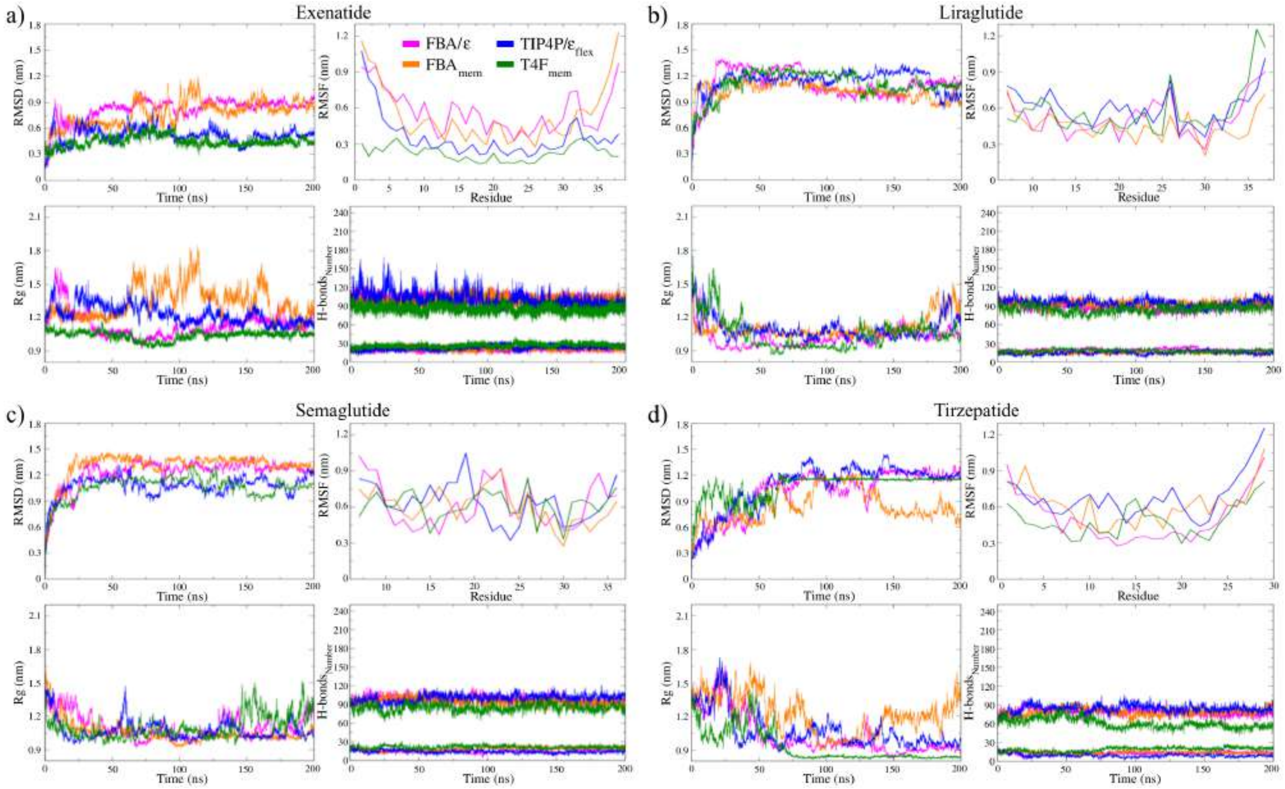

Figure S2. Helical peptides used to assess the LEw models and their stability indicators obtained from the different MD trajectories for a) Exenatide, b) Liraglutide, c) Semaglutide, and d) Tirzepatide. The same color code as in the manuscript was used in all graphs: orange and green for the LEw models (FBA_{mem} and $T4F_{mem}$, respectively), and magenta and blue for the FBA/ϵ and TIP4P/ϵ_{flex} models.

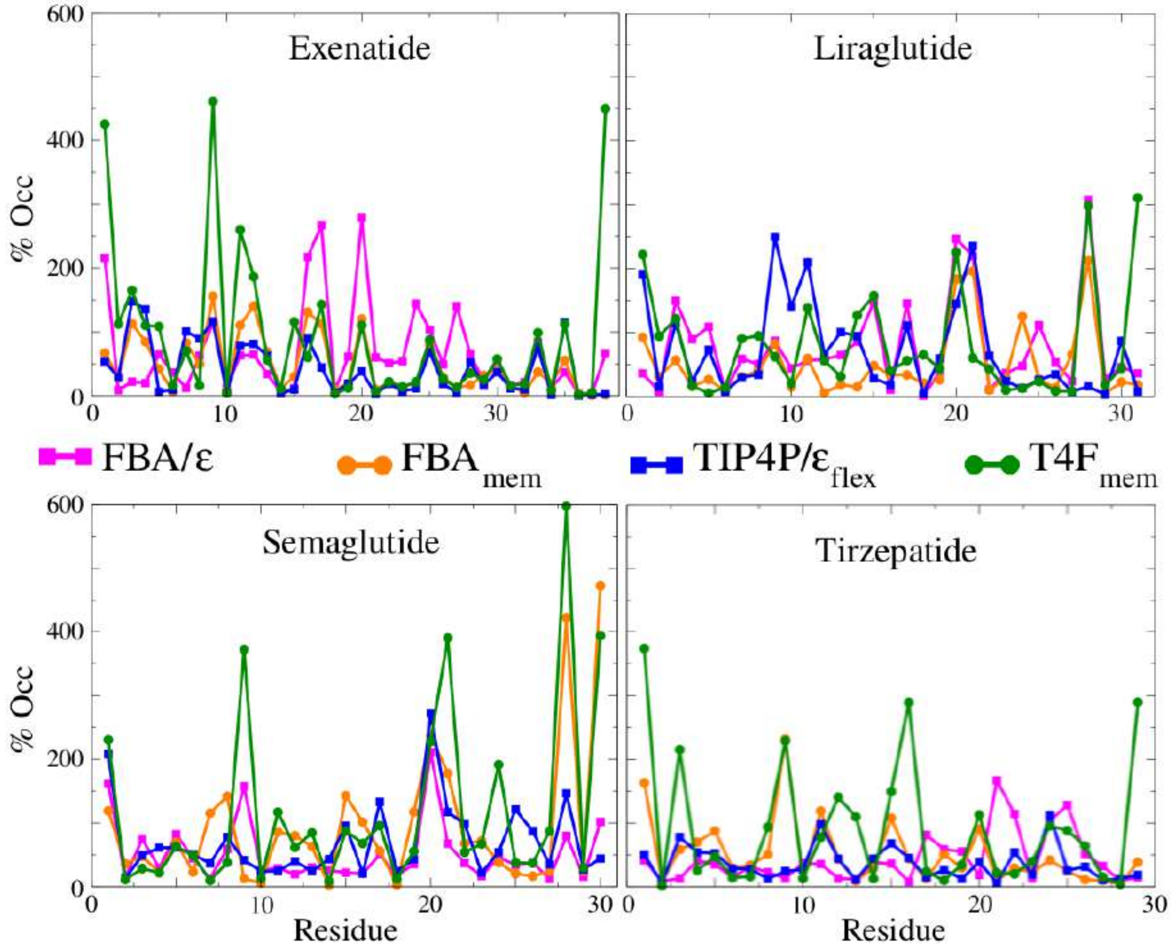

Figure S3. Occupancy percentages obtained along the MD trajectories. The reported values include intramolecular and peptide-water interactions. Values greater than 100% indicate multiple interactions of the analyzed peptide. The same color coding is used to illustrate the different water models employed in the present work.

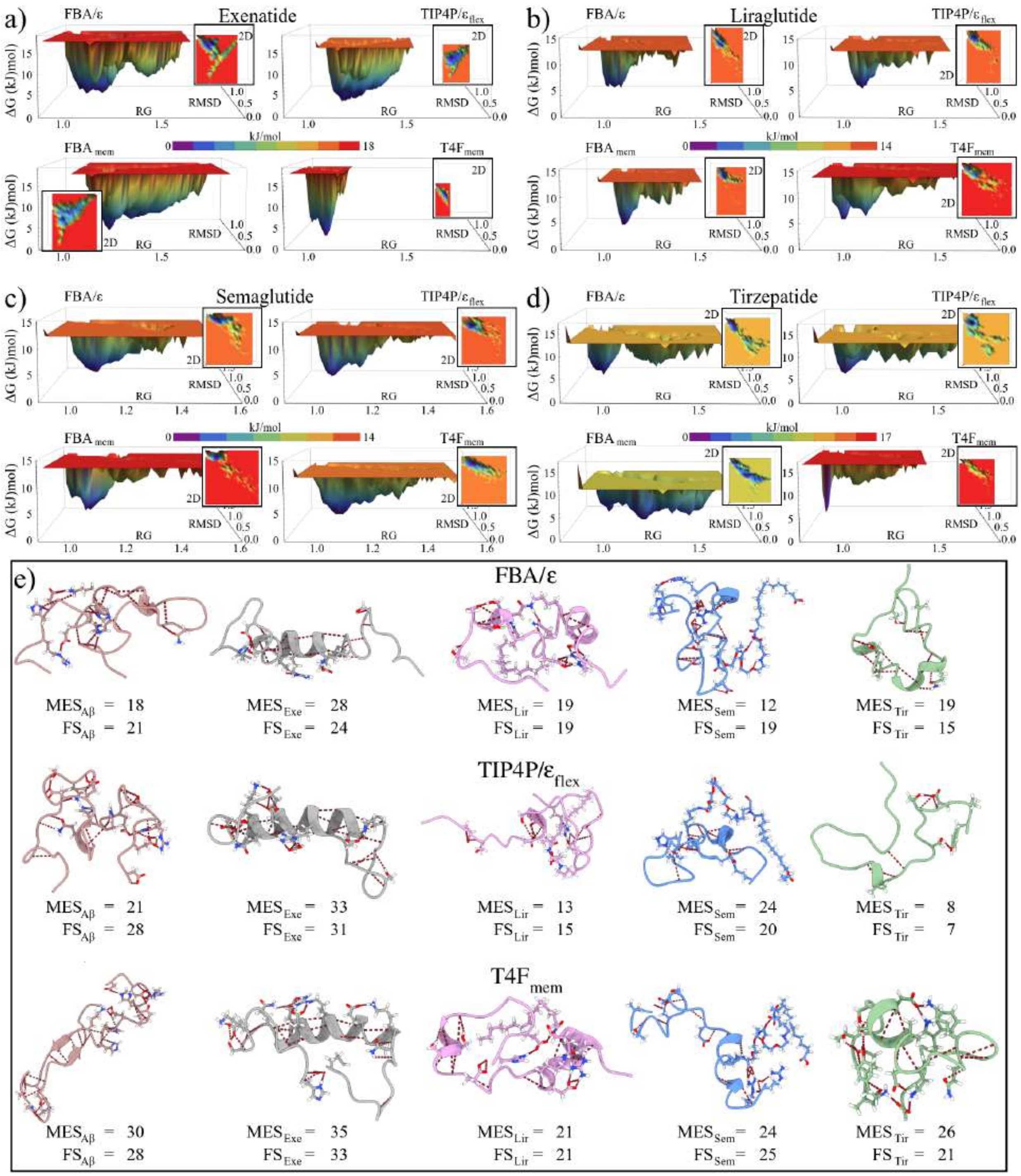

Figure S4. Analysis of the free energy landscape (FEL) and intramolecular hydrogen bonds of the helical peptides used in this work. a-d) Configurational spaces obtained using the stability indicators RMSD and radius of gyration as variables in the calculation of energies during the complete MD trajectories. The insets are the top-down views of the 3D plots. For a more accurate comparison of the different substates, the same axes dimensions were used in both the 3D and 2D plots. e) Intramolecular H-bonds of the minimum energy structures (obtained from the FEL analysis) and final (200 ns) for the systems solvated with the FBA/ϵ, TIP4P/ϵ_{flex}, and T4F$_{mem}$ models.

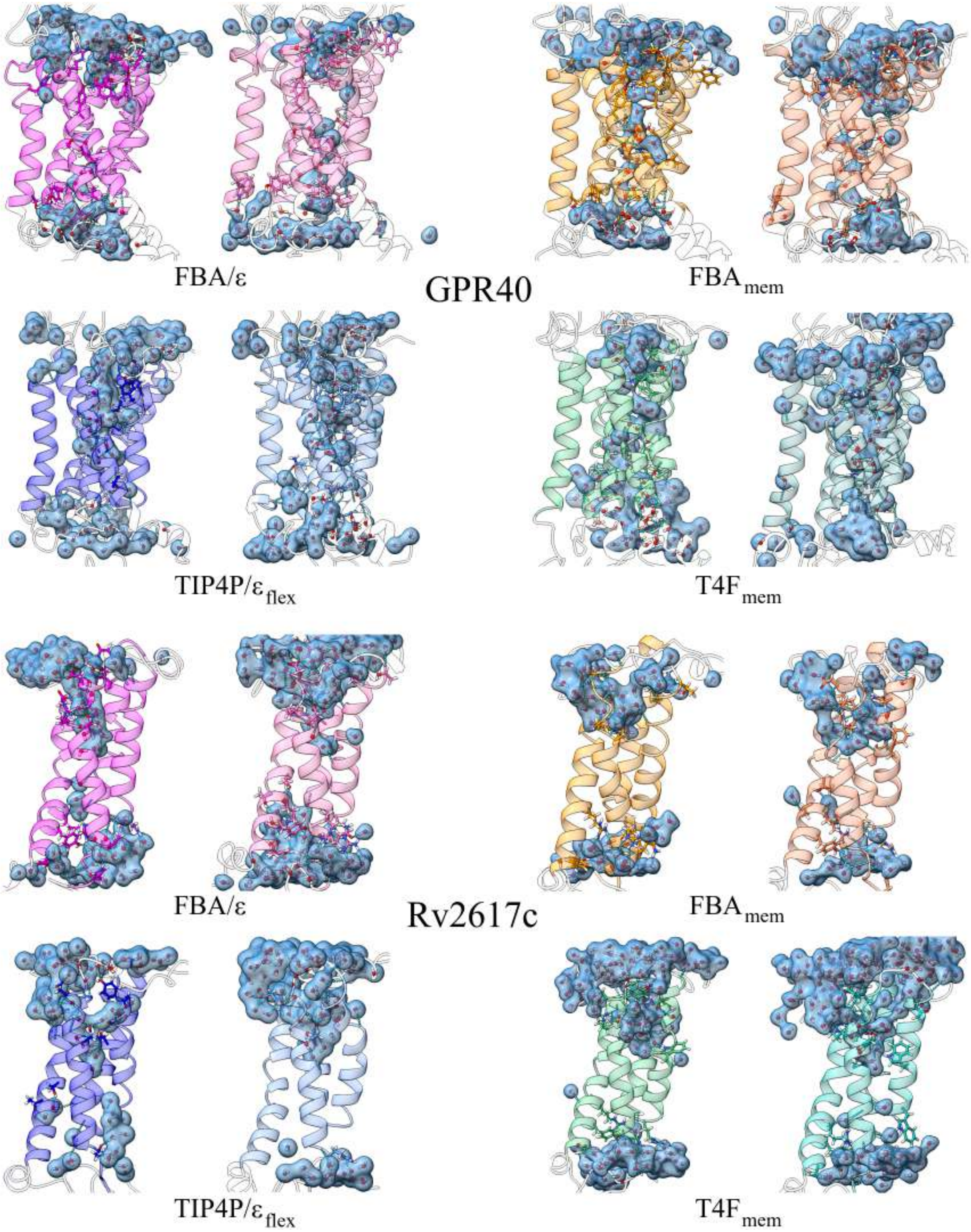

Figure S5. Penetration of water molecules into the core of membrane proteins. For each water model analyzed, the final structures of both MPs are shown, both from the original simulation and its replica. The protein structures are shown in their ribbon representation, with each model color-coded as used in the previous figures. Water molecules are shown as translucent blue surfaces. For better visualization, the membrane lipid molecules have been omitted.

Supplementary Tables

Table S1. Stability descriptors of solvated peptides.

Water Model	^{a}RMSD	^{a}RMSF	^{a}Radius of gyration	^{b}SASA	^{c}H-bonds			$^d\alpha$-helix
					Intra	Prot-solv	solv-solv	%
Aβ-42								30 (71.4)
FBA/ϵ	1.22±0.19	0.53±0.11	1.14±0.11	38.67±2.33	16±3	117±6	32698±64	6 (14.3)
FBA$_{mem}$	1.13±0.18	0.57±0.12	1.10±0.28	34.97±4.44	26±5	100±9	31691±68	3 (7.1)
TIP4P/ϵ_{flex}	1.30±0.20	0.63±0.12	1.10±0.08	38.61±2.17	17±3	115±8	32559±68	3 (7.1)
T4F$_{mem}$	1.23±0.13	0.68±0.14	1.27±0.12	39.03±2.05	22±3	99±7	28907±88	6 (14.3)
Exenatide								21 (55.3)
FBA/ϵ	0.82±0.13	0.57±0.18	1.14±0.11	34.22±1.78	20±3	102±6	12252±38	13 (34.2)
FBA$_{mem}$	0.78±0.15	0.54±0.24	1.34±0.14	37.94±2.58	22±3	100±7	11870±43	14 (36.8)
TIP4P/ϵ_{flex}	0.52±0.09	0.36±0.18	1.23±0.09	35.11±1.79	23±4	102±12	13107±492	16 (42.1)
T4F$_{mem}$	0.45±0.06	0.22±0.06	1.04±0.04	31.36±1.28	27±4	85±7	10673±54	13 (34.2)
Liraglutide								11 (35.5)
FBA/ϵ	1.11±0.17	0.53±0.16	1.02±0.08	30.71±2.33	19±3	89±6	31601±61	6 (19.4)
FBA$_{mem}$	1.03±0.10	0.45±0.12	1.11±0.09	32.81±2.44	15±2	93±6	30616±68	7 (22.6)
TIP4P/ϵ_{flex}	1.14±0.13	0.60±0.15	1.10±0.10	34.32±2.04	15±3	95±7	31436±64	3 (9.7)
T4F$_{mem}$	1.11±0.15	0.57±0.20	1.05±0.14	32.60±3.00	18±3	85±7	27917±88	8 (25.8)
Semaglutide								26 (86.7)
FBA/ϵ	1.23±0.16	0.62±0.20	1.11±0.11	35.03±2.59	15±3	99±6	29627±61	6 (20.0)
FBA$_{mem}$	1.31±0.16	0.60±0.15	1.05±0.09	35.92±2.23	19±3	95±6	28704±63	4 (13.3)
TIP4P/ϵ_{flex}	1.08±0.11	0.64±0.16	1.07±0.09	35.52±2.42	14±3	100±7	29409±64	4 (13.3)
T4F$_{mem}$	1.09±0.12	0.62±0.12	1.10±0.11	35.20±2.46	21±3	84±7	26103±91	8 (26.7)
Tirzepatide								27 (93.1)
FBA/ϵ	1.01±0.26	0.51±0.20	1.03±0.18	29.44±2.80	13±3	78±6	24251±54	13 (44.8)
FBA$_{mem}$	0.84±0.19	0.62±0.16	1.22±0.17	32.23±1.96	14±2	79±6	23496±60	9 (31.0)
TIP4P/ϵ_{flex}	1.08±0.27	0.68±0.18	1.07±0.16	30.72±2.41	9±3	84±6	23463±55	3 (10.3)
T4F$_{mem}$	1.07±0.15	0.49±0.13	0.93±0.14	26.16±3.68	19±4	61±8	20859±75	5 (17.2)

[a] Values in nanometers. [b] Values in square nanometers. [c] Number of H-bonds formed. [d] Number of residues forming the α-helix in final structures. Parentheses numbers represent their percentage over the total residues. All values were obtained from the last 100 ns of the MD trajectories.

Table S2. Average H-bond occupancy of the intramolecular interactions in the analyzed peptides. The values are expressed in percentages.

**Water model**	**Aβ-42**	**Exenatide**	**Liraglutide**	**Semaglutide**	**Tirzepatide**
FBA/ϵ	70.95±82.28	67.85±71.77	77.88±73.31	52.45±48.63	43.13±39.81
FBA$_{mem}$	116.82±99.20	47.60±44.73	50.52±56.02	93.88±111.43	50.88±51.48
TIP4P/ϵ_{flex}	62.04±76.66	41.78±42.83	71.95±71.82	69.90±59.27	36.44±26.93
TIP4P$_{mem}$	85.40±77.69	91.32±121.11	81.19±83.79	117.97±114.28	90.71±99.85

Table S3. Results of the Free Energy Landscape (FEL) analysis.

Peptide	Water Model	[a]FEL area (nm^2)	FEL energy (kJ/mol)	[b]Minimum structures	H-bond Intra	α-helix %
Aβ-42	FBA/ϵ	0.92	12.70	3	17<br>22<br>19	10(23.8)<br>7(16.6)<br>10(23.8)
	FBA$_{mem}$	1.43	18.50	1	28	6(14.3)
	TIP4P/ϵ_{flex}	1.06	12.60	1	14	7(16.6)
	TIP4P$_{mem}$	1.11	11.60	1	30	0(0)
Exenatide	FBA/ϵ	0.74	17.90	2	27<br>26	17(44.7)<br>9(23.7)
	FBA$_{mem}$	0.92	18.00	1	38	18(47.4)
	TIP4P/ϵ_{flex}	0.40	16.40	2	35<br>35	17(44.7)<br>18(47.4)
	TIP4P$_{mem}$	0.18	18.00	3	40<br>37<br>41	17(44.7)<br>11(28.9)<br>13(34.2)
Liraglutide	FBA/ϵ	0.79	12.80	1	18	4(13.3)
	FBA$_{mem}$	0.63	12.90	2	18<br>15	3(10.0)<br>4(13.3)
	TIP4P/ϵ_{flex}	0.80	12.80	1	16	4(13.3)
	TIP4P$_{mem}$	1.15	14.00	2	22<br>22	3(10.0)<br>9(30.0)
Semaglutide	FBA/ϵ	0.83	12.40	2	17<br>24	8(27.6)<br>7(24.1)
	FBA$_{mem}$	0.97	13.80	2	19<br>28	4(13.8)<br>7(24.1)
	TIP4P/ϵ_{flex}	0.73	12.70	1	14	4(13.8)
	TIP4P$_{mem}$	0.76	12.00	4	29<br>30<br>33<br>33	10(34.5)<br>6(20.7)<br>4(13.8)<br>3(10.3)
Tirzepatide	FBA/ϵ	1.06	12.90	2	15<br>14	10(34.5)<br>17(58.6)
	FBA$_{mem}$	0.97	11.20	1	17	7(24.1)
	TIP4P/ϵ_{flex}	1.20	13.10	1	9	0(0.0)
	TIP4P$_{mem}$	0.74	16.80	2	29<br>23	7(24.1)<br>7(24.1)

[a] Values in square nanometers. [b] Number of energy minima in configurational space obtained from FEL analysis.

Table S4. Average number of H-bonds formed among the TM domains of GPR40 and Rv2617c proteins during MD trajectories.

Water model	Transmembrane domains							Total	TM - water
	**TM1**	**TM2**	**TM3**	**TM4**	**TM5**	**TM6**	**TM7**	**H-bonds**	**Interactions**
**GPR40 protein**									
FBA/ϵ r1	1.11±0.80	4.24±0.81	3.67±1.00	2.02±0.65	2.28±1.20	4.90±1.66	2.25±1.04	20.47	50.97
FBA/ϵ r2	1.31±0.73	4.51±0.91	2.92±1.27	2.23±0.52	3.20±0.91	5.06±1.26	3.49±1.21	22.72	57.07
FBA_{mem} r1	1.88±1.02	3.89±0.91	2.33±0.80	2.56±0.57	2.48±0.78	5.28±1.15	4.10±1.27	22.52	65.24
FBA_{mem} r2	1.38±0.84	4.14±0.89	1.43±0.73	1.24±0.62	1.24±1.07	2.20±0.37	2.24±1.09	13.87	75.30
TIP4P/ϵ_{flex} r1	0.70±0.47	3.33±0.89	2.85±1.00	2.95±0.77	4.18±1.50	2.75±1.38	4.26±1.39	21.02	101.36
TIP4P/ϵ_{flex} r2	0.69±0.68	3.10±1.57	3.13±1.06	1.88±0.90	2.77±0.93	5.21±1.48	3.74±2.09	20.52	89.52
$T4F_{mem}$ r1	1.17±0.68	3.86±0.70	1.95±0.95	1.88±0.46	4.21±1.05	4.41±1.26	1.95±1.12	19.43	72.31
$T4F_{mem}$ r2	1.17±0.70	3.46±0.98	1.28±0.96	2.04±0.37	5.39±1.11	6.03±1.39	1.91±0.99	21.28	79.32
**Rv2617c protein**									
FBA/ϵ r1	3.01±0.69	3.62±0.82	1.44±0.63	1.03±0.74	-	-	-	9.10	62.70
FBA/ϵ r2	3.34±1.01	2.07±1.11	1.01±0.98	1.43±0.83	-	-	-	7.85	69.22
FBA_{mem} r1	3.32±0.89	3.44±0.94	1.26±0.80	1.61±0.97	-	-	-	9.63	66.45
FBA_{mem} r2	2.73±1.49	3.14±0.99	0.76±0.85	1.57±1.15	-	-	-	8.20	64.14
TIP4P/ϵ_{flex} r1	2.89±0.85	1.89±0.73	1.13±0.99	1.52±0.93	-	-	-	7.43	70.25
TIP4P/ϵ_{flex} r2	2.79±1.11	2.56±0.94	0.48±0.56	0.71±0.79	-	-	-	6.54	62.53
$T4F_{mem}$ r1	4.51±1.07	2.51±0.77	1.10±0.68	3.10±0.97	-	-	-	11.22	64.97
$T4F_{mem}$ r2	4.40±1.07	2.68±0.72	1.26±0.67	2.98±1.09	-	-	-	11.32	67.11

Table S5. Top ten residues with the highest occupancy in the MP-water interactions.

No.	FBA/ϵ		FBA_{mem}		TIP4P/ϵ_{flex}		$T4F_{mem}$	
	MP-w	TM-w	MP-w	TM-w	MP-w	TM-w	MP-w	TM-w
GPR40								
1	E145 (750.2)	R183 (381.2)	E145 (599.5)	R183 (379.5)	D152 (1445.9)	D52 (1040.4)	K300 (1146.2)	D52 (910.1)
2	K300 (748.2)	R28 (249.8)	K300 (594.7)	D52 (294.5)	D2 (1426.5)	Y240 (445.1)	D2 (1112.4)	R183 (590.7)
3	D2 (653.2)	D52 (246.0)	D2 (575.8)	Y240 (236.9)	E145 (1380.9)	K259 (428.6)	E65 (930.7)	R258 (498.4)
4	E172 (557.8)	K259 (242.8)	E172 (537.3)	R258 (224.8)	K300 (1238.1)	R28 (425.2)	E172 (890.0)	R28 (480.6)
5	D175 (534.4)	R258 (217.0)	D152 (505.4)	R28 (217.7)	E172 (1163.1)	R183 (424.1)	D175 (877.4)	Y240 (438.2)
6	D152 (521.3)	Y240 (210.5)	D175 (491.4)	K259 (203.3)	D175 (1163.0)	H86 (420.5)	E145 (830.7)	Y278 (378.6)
7	R292 (437.8)	Y278 (171.2)	R292 (432.7)	S247 (187.9)	E65 (996.2)	R258 (352.4)	H153 (826.3)	S247 (337.7)
8	E65 (420.7)	K62 (160.6)	N165 (421.6)	H86 (177.9)	N165 (785.6)	Y278 (346.3)	D152 (714.8)	Y12 (334.4)
9	R118 (407.4)	S247 (160.5)	E65 (405.8)	N244 (177.2)	R292 (732.5)	N244 (335.4)	N155 (699.9)	K62 (326.7)
10	N165 (400.3)	T276 (113.7)	N155 (384.1)	Y278 (176.4)	R211 (724.2)	N241 (305.4)	R104 (696.3)	H86 (323.3)
R2617c								
1	D62 (702.8)	D69 (698.8)	D62 (573.8)	D69 (559.6)	D62 (1430.8)	D69 (1366.9)	D62 (998.5)	D116 (1003.8)
2	P146 (636.0)	D116 (610.6)	P146 (401.3)	D116 (517.4)	P146 (1366.9)	D116 (1184.9)	P146 (938.3)	D69 (988.3)
3	D13 (552.8)	D121 (481.0)	D13 (397.7)	D121 (509.3)	D13 (1118.4)	D121 (843.5)	D13 (727.2)	E79 (872.2)
4	R4 (340.2)	E79 (429.7)	R4 (376.4)	E79 (360.6)	D17 (795.3)	E79 (800.5)	R4 (641.6)	D121 (858.9)
5	R145 (325.6)	H139 (256.2)	D17 (325.3)	S140 (243.4)	R4 (652.2)	D40 (748.8)	H48 (580.8)	S140 (511.0)
6	N61 (290.6)	D40 (248.9)	R145 (321.1)	R91 (241.3)	R145 (616.1)	R120 (560.0)	N53 (575.7)	H139 (434.6)
7	D17 (258.2)	S140 (247.4)	N53 (299.6)	R120 (225.7)	N53 (560.5)	S140 (524.1)	H51 (556.4)	R27 (417.4)
8	Q50 (253.8)	R120 (246.4)	Q50 (286.9)	H139 (214.7)	N61 (541.1)	H139 (502.2)	T6 (531.7)	Q136 (386.3)
9	Y55 (215.7)	R91 (231.5)	T6 (273.4)	R27 (185.9)	H51 (531.3)	R91 (426.7)	Q50 (492.4)	R133 (382.1)
10	K16 (203.2)	R133 (211.6)	T7 (260.1)	K41 (184.3)	Q50 (505.9)	Q136 (330.6)	T7 (489.7)	R120 (361.1)

Values in parentheses are the percentage of occupancy of the H-bond interactions over the complete trajectories of the DM simulations.

Table S6. Electrostatic interactions with significant occupancy values.

GPR40	F9–A63	N23–G49	N23–D52	Y91–N241	W224–L274	S243–R258	Significant interactions
FBA/ϵ	22.8	30.7	46.8	36.4	41.1	23.0	D47-W124 (42.6), Y91-N244 (41.0), S101-V225 (33.6), S101-A229 (33.9), H137-A182 (42.6), L235-W267 (32.0)
FBA_{mem}	23.5	41.6	50.3	45.4	40.8	10.5	N47-W124 (41.9), S101-A226 (46.3), S187-N241 (34.8)
TIP4P/ϵ_{flex}	10.2	9.5	8.7	33.2	52.5	17.6	K62-R258 (41.1), Y91-H137 (41.6), Y91-S187 (67.3), S101-T198 (44.1), S101-V225 (52.9), R183-S247 (57.1), S187-F248 (37.9)
$T4F_{mem}$	27.6	50.2	7.9	37.5	13.2	10.3	Y91-Y240 (34.7), R183-N244 (102.6), L186-N241 (50.6)
Rv2617c	R27–V86	F30–L124	D40–R120	E79–W100	R91–Q136	- -	Significant interactions
FBA/ϵ	49.8	9.9	45.9	92.3	21.4		
FBA_{mem}	29.3	10.7	29.5	62.3	14.9		Y23-H139 (113.0), E79-R120 (42.9)
TIP4P/ϵ_{flex}	12.3	12.1	37.1	50.0	5.7		K41-D121 (119.5)
$T4F_{mem}$	9.9	14.5	89.8	89.9	16.4		K41-D121 (116.4)

The values in parentheses correspond to the H-bond occupancy as a percentage over the entire MD trajectory.
